# Emerging neuroprotective mechanisms and therapeutic potential of natural polysaccharides in Alzheimer's disease

**DOI:** 10.3389/fnagi.2026.1877358

**Published:** 2026-08-04

**Authors:** Noor Muhammad, Rui Ai, Limin Yang, Yuelong Cui, Liqiang Wang, Shaoting Weng, Yao Wang, Mohib Ullah Kakar, Suliman Khan, Sajjad Ahmad, Imran Iqbal

**Affiliations:** 1 College of Biology and Food Engineering, Anyang Institute of Technology, Anyang, China; 2 College of Life Sciences, Jinggangshan University, Ji'an, China; 3 Biomedical Sciences, Radiation Therapy Department, Anyang District Hospital, Puyang, China; 4 Faculty of Marine Sciences, Lasbela University of Agriculture Water and Marine Sciences (LUAWMS), Uthal, Balochistan, Pakistan; 5 Faculty of Veterinary and Animal Sciences, Lasbela University of Agriculture, Water and Marine Science, Uthal, Baluchistan, Pakistan; 6 Department of Pathology, NYU Grossman School of Medicine, New York University Langone Health, New York, NY, United States; 7 Department of Surgery - Otolaryngology, School of Medicine, Yale University, New Haven, CT, United States

**Keywords:** Alzheimer's disease (AD), neuroinflammation, neuroprotection, polysaccharides, tau protein

## Abstract

Alzheimer's disease (AD) is a progressive neurodegenerative disorder characterized by cognitive decline and memory loss, affecting millions worldwide. Despite extensive research, effective treatments remain limited. Polysaccharides, abundant biological macromolecules derived from natural resources have emerged as promising therapeutic candidates due to their antioxidant, anti-inflammatory, and neuroprotective properties. This narrative review summarizes recent preclinical and limited clinical evidence on natural polysaccharides and their structure–activity relationships (SAR) in AD. We focus on their ability to mitigate amyloid-beta (Aβ) aggregation, reduce tau hyperphosphorylation, modulate neuroinflammation, and enhance cognitive function. We emphasize novel mechanistic insights and highlight how fungal, algal, plant, and bacterial polysaccharides exert broad multi-targeted effects. Importantly, this is the first review to integrate SAR with challenges of bioavailability and blood–brain barrier (BBB) penetration, offering a framework for optimizing therapeutic design. Novel insights into the molecular mechanisms, such as activation of Nrf2 and autophagy pathways, are discussed. We also underscore the translational value of polysaccharides, noting that strategies such as structural modification, nanoparticle conjugation, and pharmacokinetic profiling could accelerate their progression toward clinical application. However, most evidence is limited to *in vitro* and animal models, with critical challenges including poor bioavailability, limited blood-brain barrier penetration, and a lack of standardized clinical data. We conclude that natural polysaccharides are promising, low-toxicity therapeutic agents for AD management. But for complete validation of their therapeutic potential, standardized pharmacokinetic profiling, rigorous safety assessment, and well-designed clinical trials are urgently required.

## Introduction

Polysaccharides, a type of abundant biological macromolecules, have been at center of attention in recent research due to their therapeutic potential in neurodegenerative disorders, including Alzheimer's disease (AD) (Mohammed et al., 2021). These complex carbohydrates, derived from diverse natural sources such as microorganisms (e.g., fungi, bacteria, algae), plants, and animals (Wang et al., 2016), demonstrate a wide range of biological activities and neuroprotective effects (Zhong et al., 2019). Notably, polysaccharides from marine algae, such as alginates, agar, and carrageenan, have been studied for their therapeutic efficacy and applications in drug delivery as well as pharmaceutical formulations (Tønnesen and Karlsen, 2002; Cherng, 2018). In the context of AD, a progressive neurodegenerative disorder characterized by cognitive decline and memory loss, natural polysaccharides have shown potential in targeting key pathological processes (Manlusoc et al., 2019; Olasehinde et al., 2019a,b, 2020; Zhong et al., 2020a). Polysaccharides have diverse applications as previously reported in cosmetic, nutraceutical, and pharmaceutical industries. Certain therapeutic effects exhibited by natural polysaccharides (among those of bacterial origin) include anticholinesterase (Olasehinde et al., 2019a,b; Mahmoud et al., 2023; Selim et al., 2023), anti-neuroinflammatory (Zhong et al., 2020b; Yu et al., 2024; Lu et al., 2025), anti-neurotoxicological (Manlusoc et al., 2019; Olasehinde et al., 2020; Zhang Z. et al., 2020; Zhang et al., 2023; Hou et al., 2024), and anti-amyloidogenic (Bai et al., 2019; Manlusoc et al., 2019; Olasehinde et al., 2019b; Zhong et al., 2020b; Makshakova et al., 2023; Zhang et al., 2023) effects making them potential broad spectrum agents for AD management.

Alzheimer's disease accounts for 60%−80% of dementia cases worldwide and affects more than 50 million people, a number projected to triple by 2050, highlighting its significant societal and healthcare burden (Nichols et al., 2022; Tahami Monfared et al., 2022; Guo et al., 2023; Xiaopeng et al., 2025; Xu et al., 2025). In AD and other NDDs, the accumulation of abnormal proteins, including amyloid-beta (Aβ) and hyperphosphorylated tau, disrupts cellular homeostasis, resulting in neuronal dysfunction and death (Guix, 2020). Early-onset AD (EOAD), occurring before the age of 65, is linked to dominant genetic mutations in *PSEN1, PSEN2*, and *APP* genes, on chromosomes 14, 1, and 21, respectively (Rostagno et al., 2009; Long and Holtzman, 2019), while late-onset AD (LOAD) which is the more common form (this accounts for 90–95% of cases and manifests after age 65) is associated with genetic risk factors like the *APOE4* gene, present in 16% of the population, as well as non-genetic factors such as cerebrovascular disease, diabetes, and poor diet (Karch et al., 2014; Long and Holtzman, 2019; Hrelia et al., 2020; Siddappaji and Gopal, 2021). These multifactorial pathological processes highlight the urgent need for finding novel therapeutic strategies (Ganguly et al., 2017).

Despite extensive research, effective treatments for AD remain limited (owing to the gradual and insidious onset of the disease, its prolonged latent period, and multifaceted pathology), which requires the development of innovative therapeutic approaches. Although recent phase 3 trials of lecanemab and donanemab have shown disease–modifying effects, the overall failure rate of AD drug development remains extremely high, with most previous anti-amyloid and anti-tau therapies failing to achieve meaningful clinical benefit despite improvements in biomarker development (Boxer and Sperling, 2023; Huang et al., 2023). Current therapies, including cholinesterase inhibitors and memantine, provide modest symptomatic relief but do not prevent disease progression, highlighting the urgent need for novel multi-targeted therapeutic approaches (Cummings, 2021; Xu et al., 2021; Xiao and Zhang, 2024). Compared to synthetic compounds, natural polysaccharides offer several advantages as therapeutic agents for AD. They generally exhibit lesser toxicity and good biocompatibility, reducing the risk of adverse effects associated with their long-term use (Feng and Hao, 2024). Many polysaccharides or their low-molecular-weight derivatives can cross the blood–brain barrier (BBB) permeability or modulate the permeability, enabling delivery to the CNS (Makani et al., 2016; Jia et al., 2022; Lu et al., 2024). These molecules show diverse mechanisms such as anti-inflammatory, antioxidant, and anti-amyloidogenic effects which provide a broad therapeutic effect compared to synthetic single-target agents (Behrad et al., 2024; Prabha et al., 2025). Their structural diversity allows for chemical modification or nanoparticle conjugation to enhance pharmacokinetics (PK) and brain delivery while maintaining safety and bioactivity (Yousfan et al., 2024; Dargude et al., 2025).

Compared to other natural compounds such as flavonoids and alkaloids, polysaccharides provide distinct advantages. While flavonoids and alkaloids demonstrate free radical scavenging and neuroprotective effects, their actions are often confined to specific molecular pathways (Meng et al., 2025; Nahar et al., 2025). In contrast, polysaccharides exert broad effects across amyloid-related pathways, oxidative stress, and neuroinflammatory cascades simultaneously, as demonstrated by their ability to inhibit amyloid-beta aggregation, enhance free radical scavenging enzyme activities, and modulate neuroinflammation (Yuchen-Zhang et al., 2024). Their structural complexity and modifiability also allow for unique strategies such as sulfation, methylation, or nanoparticle conjugation, offering enhanced pharmacokinetics and brain delivery that other natural bioactives cannot achieve as effectively (Barclay et al., 2019; Cao et al., 2024)

Natural polysaccharides, derived from diverse sources such as fungi (e.g., *Hericium coralloides*, and *Cantharellus cibarius*), algae (e.g., *Ulva lactuca*, and *Ecklonia maxima*), and plants (e.g., *Astragalus membranaceus*, and *Dendrobium officinale*), have been extensively studied for their therapeutic potential, particularly plant-derived polysaccharides (Kakar et al., 2020b, 2022; Xiao et al., 2024). These macromolecules exhibit multi-targeted effects, including inhibition of Aβ aggregation, reduction of tau hyperphosphorylation, modulation of neuroinflammation, and activation of pathways such as Nrf2 and autophagy, as evidenced in preclinical AD models (Zhang X. et al., 2022). Given the diversity of evidence and limited number of clinical studies, a narrative review approach was adopted to summarize and critically evaluate the current knowledge. This review discusses recent evidence on the neuroprotective mechanisms and structure-activity relationships of polysaccharides from fungal, algal, and plant sources, evaluates their efficacy in *in vivo* and *in vitro* studies, and addresses challenges in bioavailability and clinical translation. Despite this progress, no review has systematically integrated structure–activity relationships (SAR), bioavailability barriers, and translational gaps of polysaccharides in AD. This review specifically addresses that knowledge gap by synthesizing mechanistic, structural, and preclinical evidence, while outlining strategies to facilitate clinical translation.

### Methods

**Figure 1:**
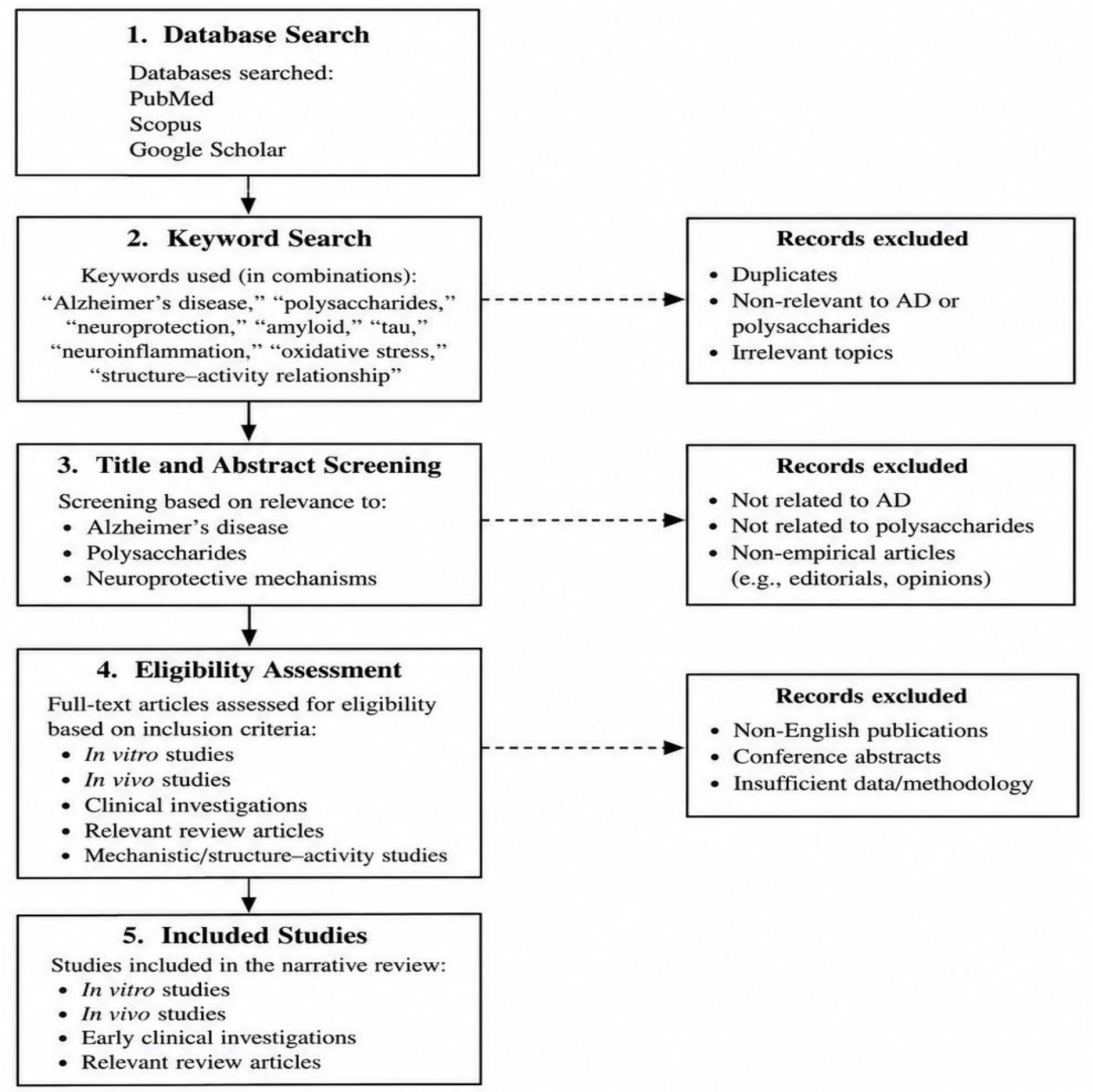
Literature identification, screening, and selection process used in this narrative review.

This work is a narrative review, developed through a structured literature search. Different databases, PubMed, Scopus, and Google Scholar were explored using combinations of the keywords: “Alzheimer's disease,” “polysaccharides,” “neuroprotection,” “amyloid,” “tau,” “neuroinflammation,” “oxidative stress,” and “structure–activity relationship.” Publications from January 2010 to June 2025 were considered, with earlier landmark studies included when directly relevant. Eligible studies included *in vitro, in vivo*, and early clinical investigations relevant to polysaccharides and AD. Review papers where relevant were included. Non-English publications, editorials, and conference abstracts were excluded. Reference lists of included articles were also screened to capture additional relevant studies. The goal was to synthesize the most relevant and recent findings to provide a comprehensive and critical overview rather than a systematic meta-analysis. A schematic overview of literature identification and selection is provided in Figure 1. The inclusion and exclusion criteria are also summarized below in Table 1.

**Table 1:** Literature selection criteria used in this narrative review.

Inclusion criteria	Exclusion criteria
Alzheimer's disease-related studies	Non-AD neurological disorders
Natural polysaccharides and derivatives	Non-polysaccharide compounds
*In vitro* studies	Conference abstracts
*In vivo* studies	Editorials
Clinical investigations	Non-English publications
Mechanistic studies	Insufficiently documented reports, preprints
Peer-reviewed review articles	Duplicated publications

### Evidence appraisal

For each cited study, we indicate the model type (*in vitro*, animal, or clinical), report sample size where available (and write NR when not reported), and categorize the strength of evidence as strong (≥2 independent *in vivo* AD studies with behavioral outcomes and convergent biomarkers), moderate (≥1 *in vivo* AD study or ≥2 convergent *in vitro* studies), or preliminary (single *in vitro* or exploratory findings). This framework is consistently applied across the Results section and summarized in the consolidated evidence table.

## Pathogenesis of AD

**Figure 2:**
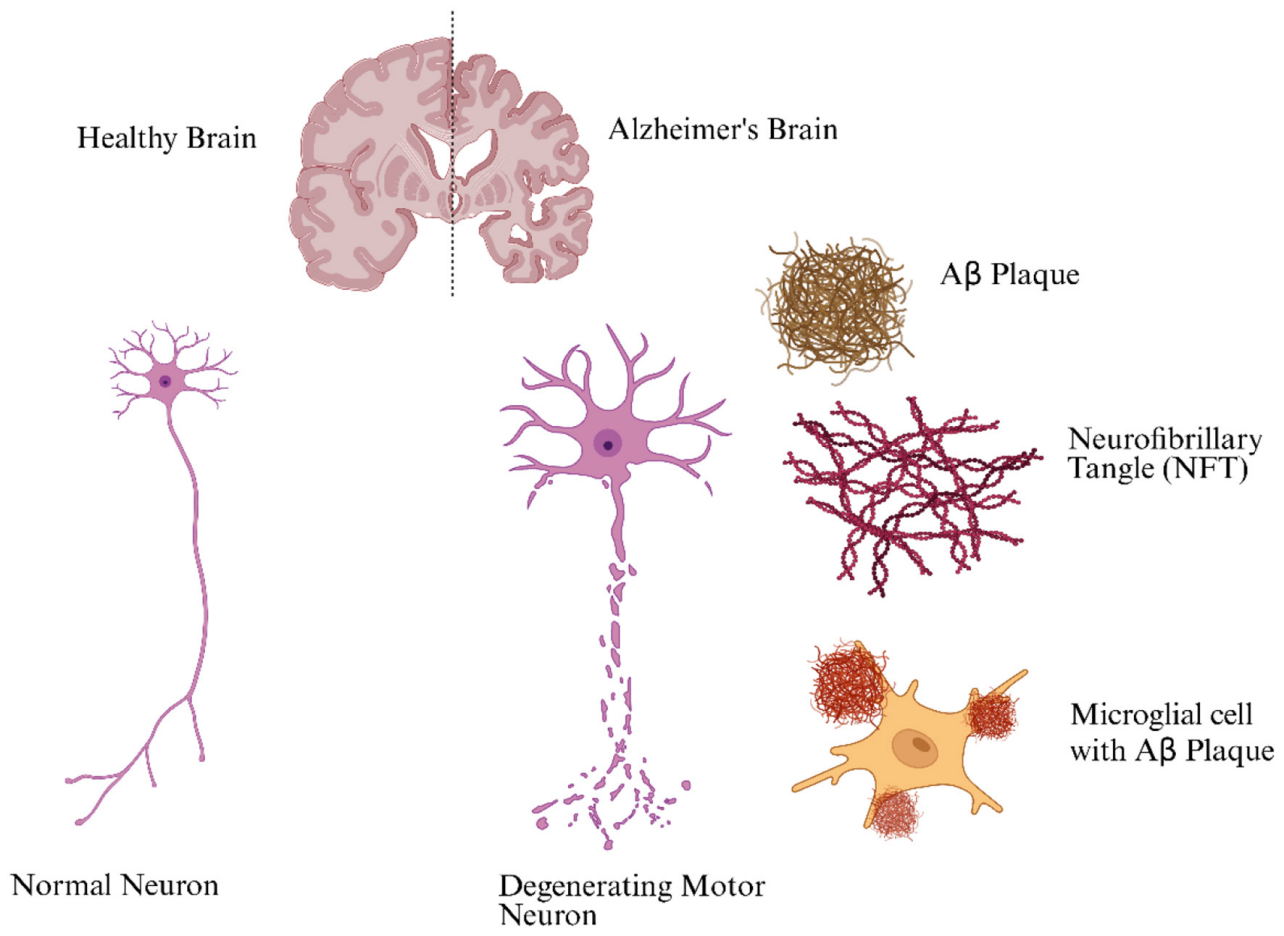
Comparative illustration of a healthy brain and Alzheimer's brain showing pathological features.

The pathological features involve hippocampal atrophy, deepened cerebral sulci, and histopathological features such as cholinergic neuronal loss in the basal forebrain, glial cell proliferation, and neuroinflammatory patches. The pathological mechanisms have been described in the previous section above. Figure 2 below summarizes the pathogenesis of AD.

**Figure 3:**
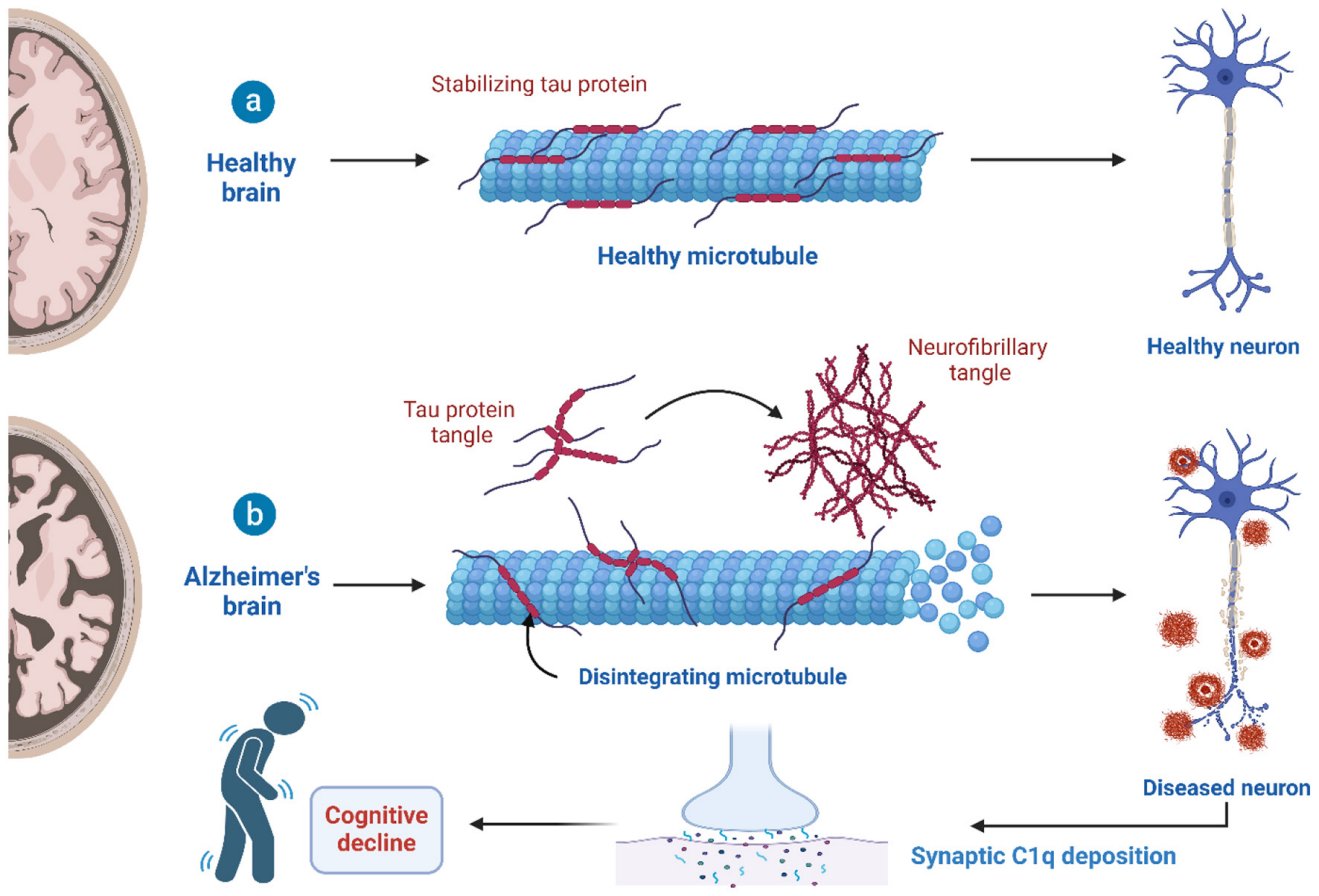
Pathology of AD showing tau protein stabilizing microtubules in healthy brain and tau tangle formation in Alzheimer's brain, leading to disintegration of microtubules and neuron damage.

In this disease, there is a chronic neurodegeneration process that continues slowly over an extended time period; therefore, it is necessary to determine a causal relationship between neurite dystrophy, neuronal apoptosis, and AD (Krasemann et al., 2017). Different *in vivo* studies using mouse models demonstrate that Aβ plaques promote tau propagation, a process that can be inhibited by tau antibodies (Dai et al., 2018; Bassil et al., 2020). However, radiological studies such as positron emission tomography (PET) with amyloid ligands, have shown that amyloid precursors may be absent in brain tissues with certain familial mutations (for example, Osaka and Arctic), suggesting that amyloid load alone does not fully account for pathology of AD (Schöll et al., 2012). In contrast, the current evidence from reported studies indicates that tau pathology [particularly neurofibrillary tangle (NFT) burden and its cortical distribution] has a much closer relationship with neuronal loss, disease progression, and cognitive decline than amyloid plaque deposition. This relationship remains complex and is influenced by neuroinflammation, synaptic dysfunction, vascular pathology, and other coexisting mechanisms (Jack et al., 2024). Current studies further support that AD arises from the complex interplay among amyloid precursor protein (APP) processing, Aβ aggregation, tau hyperphosphorylation, neuroinflammation, oxidative stress, and genetic susceptibility rather than a single pathogenic mechanism (Gholami, 2023). Figure 3 below summarizes the pathological features of Alzheimer's, including the formation of NFTs. Neuroinflammation, driven by activated microglia and astrocytes, plays a critical role in AD progression. Microglia, functioning as innate immune and phagocytic cells, maintain homeostasis and neuroprotection but contribute to neuronal damage when overactivated, correlating with increased amyloid load (Edison et al., 2008). Proinflammatory A1 astrocytes, induced by microglia, secrete cytokines such as IL-1 (interleukin-1), C1q (complement component 1q), and TNF (tumor necrosis factor) through NF-κB (nuclear factor kappa-light-chain-enhancer of activated B cells)-dependent pathways, exacerbating neuronal and oligodendrocyte damage (Liddelow et al., 2017). In contrast, M2 microglia induce anti-inflammatory astrocytes that promote cytokine release through STAT6 (signal transducer and activator of transcription 6) signaling, mitigating inflammation. These astrocytes, in turn, secrete neurotrophic factors and reduce the production of proinflammatory mediators, thereby mitigating neuroinflammation and limiting neuronal damage in AD models (Liddelow et al., 2017; Xie et al., 2020). These findings highlight that neither Aβ plaques nor NFTs alone explain AD pathogenesis, emphasizing the existence of interplay involving neuroinflammation and protein misfolding.

Current pharmacological management of AD depends primarily on cholinesterase inhibitors and the NMDA receptor antagonist memantine, which provide symptomatic relief but do not halt disease progression (Mangialasche et al., 2010; Liu et al., 2018). More recently, anti-amyloid monoclonal antibodies (mAbs) such as aducanumab and lecanemab have demonstrated disease-modifying effects in selected patients with early AD, although important limitations remain (Shi et al., 2022; van Dyck et al., 2023). These limitations have stimulated interest in multi-target therapeutic strategies. The sections below discuss recent research on polysaccharides as potential AD therapeutics, emphasizing the need for further studies to elucidate their neuroprotective mechanisms. Table 2 below summarizes mechanisms, sources, models, and evidence strength of natural polysaccharides in AD pathogenesis.

**Table 2:** Mechanisms, sources, models, and evidence strength of natural polysaccharides in Alzheimer's disease.

Pathological target	Effect on AD	Representative polysaccharide (source)	Experimental models	Sample size	Evidence tier	References
Amyloid-beta (Aβ)	Aβ accumulation impairs synaptic function and promotes neuronal death	*Hericium coralloides* polysaccharides (fungus), Alginate MOS (algae), *Astragalus membranaceus* APS (plant)	APP/PS1 transgenic mice; *Drosophila* AD models; neuronal cell lines	Rodents: *n* = 8–10; *Drosophila*/cell studies: not reported	Moderate (rodent studies); Preliminary (*Drosophila*/cell studies)	Galimberti and Scarpini, 2012; Jouanne et al., 2017; Bi et al., 2018a; Olasehinde et al., 2019a; Qin et al., 2020; Hu et al., 2021b
Tau protein	Hyperphosphorylated tau disrupts cytoskeleton and axonal transport	*Dendrobium officinale* DOP (plant), *Astragalus membranaceus* APS (plant)	SAMP8 mice; *Drosophila* AD models	Not reported	Moderate (rodent studies); Preliminary (*Drosophila* studies)	Jouanne et al., 2017; Dai et al., 2018; Feng et al., 2019; Manlusoc et al., 2019; Olasehinde et al., 2019b; Qin et al., 2020
Neuroinflammation	Microglial activation and pro-inflammatory cytokines exacerbate neuronal injury	*Cantharellus cibarius* CCP (fungus), *Ulva lactuca* polysaccharides (algae), *Acanthopanax senticosus* (plant)	Rodent AD models; microglial cell lines	Rodents: *n* = 8–10; cell studies: not reported	Moderate (rodent studies); Preliminary (cell studies)	Liddelow et al., 2017; Liu et al., 2018; Olasehinde et al., 2020; Zhong et al., 2020b; Wang et al., 2022
Oxidative stress	Reactive oxygen species (ROS) damage neurons and impair antioxidant defenses	*Annona muricata* polysaccharides, ALP (plant), *Codonopsis pilosula* CPP (plant), Fucoidan (marine)	HT22, PC12 and SH-SY5Y cell lines; rodent AD models; *C. elegans* AD models	Rodents/*C. elegans*: *n* = 8–12; cell studies: not reported	Preliminary to moderate	Edison et al., 2008; Liu et al., 2018; Manlusoc et al., 2019; Olasehinde et al., 2019a; Kim et al., 2020; Sirin and Aslim, 2021; Li R. et al., 2024
Mitochondrial dysfunction	Energy metabolism impairment, ROS accumulation, and apoptosis contribute to neurodegeneration	*Acanthopanax senticosus* polysaccharides (plant), *L. plantarum* EPS (bacteria)	Rodent AD models; PC12 and SH-SY5Y cell lines	Not reported	Moderate (rodent studies); preliminary (cell studies)	Ganguly et al., 2017; Olasehinde et al., 2020; Hu et al., 2021b; Sirin, 2023
Impaired proteostasis/ autophagy	Reduced clearance of misfolded proteins aggravates Aβ and tau pathology	*Hericium erinaceus* mycelia extract (fungus), alginate MOS (algae), durian seed DSPP-1 (plant)	APP/PS1 transgenic mice; A53T mice; *C. elegans* AD models; neuronal cell lines	Rodents: *n* = 8–10; *C. elegans*/cell studies: not reported	Moderate (rodent studies); preliminary (*C. elegans*/cell studies)	Ganguly et al., 2017; Bi et al., 2018a, 2021; Olasehinde et al., 2019b; Qin et al., 2020
Cholinesterase inhibition	AChE and BChE increase cholinergic dysfunction	*Marinobacter nauticus* EPS (bacteria), *Bacillus maritimus* BMEPS (marine bacteria)	Enzymatic assays; fish models; mouse models	Fish/mice: *n* = 10; *in vitro* studies: not reported	Moderate (animal studies); preliminary (*in vitro* studies)	Gangalla et al., 2021; Selim et al., 2023; Abdel-Razik et al., 2024

Aβ, amyloid beta; APP, amyloid precursor protein; MOS, mannuronate oligosaccharide; DOP, *Dendrobium officinale* polysaccharide; APS, *Astragalus membranaceus* polysaccharide; IL-1β, interleukin-1 beta; TNF-α, tumor necrosis factor alpha; NF-κB, nuclear factor kappa-light-chain-enhancer of activated B cells; CCP, *Cantharellus cibarius* polysaccharide; ROS, reactive oxygen species; Nrf2, nuclear factor erythroid 2-related factor 2; BChE, butyrylcholinesterase; EPS, exopolysaccharide.

## Mechanistic evidence

Multiple pathological mechanisms contribute to AD, and among them, Aβ accumulation and tau pathology are the most validated. This has been shown by neuropathology, biomarker studies, and clinical trial outcomes. Amyloid biomarkers are critical to current diagnostic strategies, while tau burden has the strongest correlation with neuronal loss, disease severity, and cognitive decline (Jack et al., 2024). Genetic studies identifying risk variants in microglial genes such as TREM2, CD33, and complement receptor type 1 along with biomarker and neuropathological evidence, support the involvement of innate immune responses and microglial activation in AD pathogenesis (Leng and Edison, 2021).

Oxidative stress and mitochondrial dysfunction can act as downstream mediators that amplify Aβ-induced injury (secondary cascades). This review also presents evidence that mitochondrial dysfunction can exist independently of Aβ and serve as a primary upstream driver. It can initiate pathologic cascades by shifting APP processing toward amyloidogenesis, as supported by cybrid studies, bioenergetic alterations in peripheral non-degenerating tissues, and links between mitochondrial failure and plaque deposition (Swerdlow, 2018). Similarly, mechanisms such as Nrf2 activation, gut microbiota modulation, and cholinesterase inhibition remain promising therapeutic targets but are currently supported predominantly by preclinical studies only.

Importantly, the therapeutic potential of natural polysaccharides lies in their ability to simultaneously modulate several of these interconnected pathways. The strongest translational rationale currently exists for polysaccharides that influence amyloid pathology, tau-associated neurodegeneration, and neuroinflammatory responses. The additional effects on oxidative stress and cellular homeostasis may provide complementary neuroprotective benefits.

## Limitations of current AD models in polysaccharide research

Cell cultures, *Caenorhabditis elegans, Drosophila melanogaster*, and transgenic mouse models such as APP/PS1 mice provide useful mechanistic insights. However, they have important limitations that should be considered when interpreting therapeutic efficacy (Drummond and Wisniewski, 2017; Sasaguri et al., 2017).

APP/PS1 and related transgenic models overexpress mutant human APP and presenilin genes to reproduce familial AD amyloid pathology. However, such mutations cause only ~1% of AD cases. Most patients develop sporadic late-onset AD driven by heterogeneous genetic and environmental factors such as APOE genotype, lipid metabolism, and neuroinflammation (Drummond and Wisniewski, 2017; van der Kant et al., 2020). Therefore, amyloid-centric models do not completely reflect the multifactorial nature of human AD. Many transgenic models exhibit robust amyloid plaque deposition, still these models lack widespread tau pathology and neurodegeneration having progressive cognitive decline seen in human disease (Mullane and Williams, 2013; Sasaguri et al., 2017).

Furthermore, several agents that reduced amyloid burden or improved cognition in mouse models have failed to demonstrate meaningful clinical efficacy in humans. This highlights the limitations of direct mouse-to-human translation in AD therapeutics (Mullane and Williams, 2013; van der Kant et al., 2020). Laboratory animals are typically genetically homogeneous, maintained under controlled environmental conditions, and studied over relatively short timeframes, whereas human AD develops over decades in genetically diverse populations with numerous coexisting or related conditions.

Sporadic AD pathogenesis involves microglial senescence, chronic inflammation, and peripheral immune factors; however, animal models show transcriptomic and phenotypic differences from human microglia and call for careful consideration (Leng and Edison, 2021). Similarly, *in vitro* neuronal cultures and lower organisms such as *C. elegans* and *Drosophila* are used for mechanistic studies. Still these models cannot fully replicate human brain architecture or complexity (Drummond and Wisniewski, 2017; van der Kant et al., 2020).

The above-described limitations are particularly relevant for polysaccharide research. Many proposed mechanisms, such as immunomodulation, gut-brain axis regulation, and neuroinflammatory modulation, depend on complex host-microbiome and immune-system interactions. These are difficult to reproduce in conventional experimental models. Future studies should therefore incorporate aged animal models, human induced pluripotent stem cell-derived neuronal and glial systems, brain organoids, multicellular neuroimmune models. Finally. well-designed clinical trials are required to improve translational relevance that would better evaluate the therapeutic potential of natural polysaccharides in AD.

Small sample sizes often limit statistical power in neuroscience, especially in animal models, undermining reproducibility and increasing risks of false positive and negative results (Button et al., 2013). Furthermore, most reported studies originating from individual laboratories have not been independently replicated across multiple research groups. Behavioral outcomes, such as Morris water maze, Y-maze, and novel object recognition tests, are particularly susceptible to methodological variability which arises from differences in animal age, sex, housing conditions, testing protocols, and experimental design (Kafkafi et al., 2018). Another important factor is that statistically significant improvements do not necessarily indicate biologically relevant effects, as effect sizes and confidence intervals are infrequently reported. That is why, although the collective evidence supporting polysaccharide-mediated neuroprotection is encouraging; the overall strength of evidence would be substantially improved through larger, adequately powered studies, standardized behavioral protocols, independent validation, and more comprehensive reporting of effect sizes and reproducibility metrics.

## Therapeutic approaches over the years

Over the years, therapeutic strategies for AD have aimed to prevent cognitive impairment and memory loss, primarily targeting the cholinergic and glutamatergic systems (Davies and Maloney, 1976; Pákáski and Kálmán, 2008). The cholinergic hypothesis attributes AD progression to acetylcholine (ACh) neuron loss and reduced receptor activity (e.g., M2 muscarinic and α7/α4β2 nicotinic receptors) in brain regions like the basal forebrain, amygdala, hippocampus, and cortex (Davies and Maloney, 1976; Mash et al., 1985; Teaktong et al., 2004; Pákáski and Kálmán, 2008). Cholinesterase inhibitors, such as donepezil, rivastigmine, and galantamine, increase ACh levels by inhibiting acetylcholinesterase (AChE), thereby improving neurotransmission and cognitive function (Andrieu et al., 2015). Galantamine also suppresses proinflammatory cytokines, addressing neuroinflammation, which is a key player in AD pathology (Liu et al., 2018). The glutamatergic hypothesis targets N-methyl-D-aspartate (NMDA) receptors to prevent calcium-mediated excitotoxicity, with drugs like memantine reducing neuronal injury (Danysz et al., 2000). Clinical trials have demonstrated the efficacy of these drugs in alleviating symptoms, although these drugs do not halt the progression of disease (Bond et al., 2012; Hyde et al., 2013). Several additional approaches, including Aβ, anti-tau, and mitochondrial-targeted therapies, have shown limited success in modifying AD pathology (Mangialasche et al., 2010). Monoclonal antibodies (mAbs) targeting Aβ (e.g., aducanumab, lecanemab; donanemab in late-phase evaluation) have shown amyloid removal and modest slowing of clinical decline in early AD but require biomarker confirmation, repeated infusions, MRI monitoring, and carry Amyloid-related imaging abnormalities (ARIA) risk; moreover, the access and cost considerations are substantial (Cummings, 2021; Shi et al., 2022; Boxer and Sperling, 2023; van Dyck et al., 2023; Huang et al., 2023; Xiao and Zhang, 2024). Small-molecule programs have included BACE1 inhibitors (largely discontinued due to safety or lack of efficacy), tau aggregation inhibitors, mitochondrial/energetic agents, and neuroinflammation modulators, with mixed outcomes to date. Symptomatic therapies (acetylcholinesterase inhibitors, memantine) remain standard of care but do not halt progression (Davies and Maloney, 1976; Mash et al., 1985; Danysz et al., 2000; Teaktong et al., 2004; Pákáski and Kálmán, 2008; Bond et al., 2012; Hyde et al., 2013; Andrieu et al., 2015). Against this backdrop, polysaccharides occupy a multi-target, low-toxicity niche: preclinical data suggest concurrent modulation of Aβ/tau handling, microglial-driven neuroinflammation, redox status (Nrf2), and autophagy. Their potential roles include (i) adjuncts to disease-modifying mAbs to dampen inflammation, stress caused by free radical formation and support synaptic resilience; (ii) earlier stage or preventive strategies where chronic oral, low risk regimens are attractive; and (iii) microbiome interacting agents (for selected polysaccharides) that may influence gut-brain immune axis. Key translational hurdles such as bioavailability/BBB penetration, pharmacokinetic variability, and manufacturing standardization. These are discussed in later sections. Due to these limitations, natural polysaccharides, offer a promising alternative (Manlusoc et al., 2019; Olasehinde et al., 2019a,b, 2020; Zhong et al., 2020a).

More recently, some studies have further highlighted the neuroprotective potential of other natural compounds that act through similar mechanisms as used by polysaccharides. Alkaloids modulate oxidative stress, neuroinflammation, and cholinergic signaling through multi-target pathways which has been determined through *in vitro* and *in vivo* studies (Kuang et al., 2024; Utpal et al., 2025). Flavonoids have been reported for their antioxidant and anti-apoptotic effects via PI3K/Akt (phosphoinositide 3-kinase/protein kinase B pathway) and NF-κB signaling (El-Maraghy et al., 2023; Faysal et al., 2025b). Berberine reduces Aβ aggregation and oxidative stress through PI3K/Akt and MAPK (mitogen-activated protein kinase). modulation which has been determined through *in vivo* studies as well (Li et al., 2023a; Begh et al., 2025). Monoterpenoids are promising therapeutic compounds that demonstrate synergistic actions with other bioactive compounds, suggesting potential combination strategies with polysaccharides (Begh et al., 2025). Moreover, modulation of gut microbiota, a known target of polysaccharides, has been identified as an emerging approach to mitigate neuroinflammation in AD (Li R. et al., 2024; Faysal et al., 2025a).

## Gut-brain axis as a mediator of polysaccharide neuroprotection

Microbiota-derived metabolites from dietary fiber influence brain function indirectly through the gut-brain axis, with limited direct central accumulation (Cryan et al., 2019; Dalile et al., 2019). Fermentation of polysaccharides by gut microorganisms generates short-chain fatty acids (SCFAs), such as acetate, propionate, and butyrate. This can regulate microglial activity, strengthen intestinal barrier integrity, reduce systemic inflammation, and influence neuroimmune communication (Dalile et al., 2019; Colombo et al., 2021). Additionally, microbiota-derived metabolites may modulate vagal signaling pathways and immune responses that influence brain function and neuroinflammation (Bonaz et al., 2018). The disturbed intestinal barrier integrity permits translocation of pro-inflammatory microbial products which promote enteric inflammation and potentially contribute to central neuroinflammation in PD and MS. The supporting evidence from ALS models; AD shows enteric inflammation and dysbiosis (Pellegrini et al., 2018). Nevertheless, most evidence remains preclinical, and studies integrating microbiome profiling, metabolomics, and clinical outcomes are further required to establish causality in human AD.

## Polysaccharides and AD

**Figure 4:**
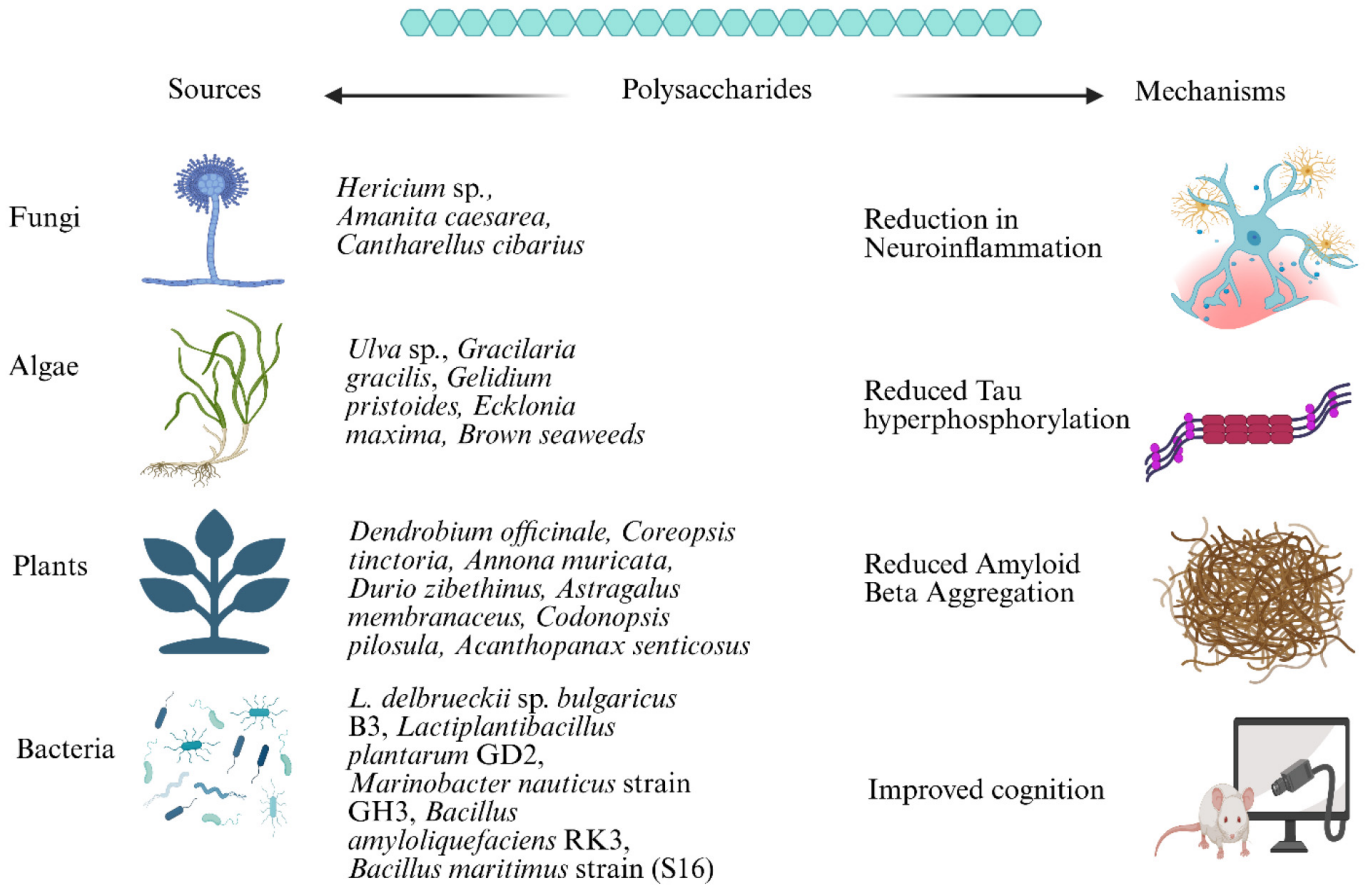
Sources of polysaccharides and their therapeutic effects for treatment of AD.

Natural polysaccharides have emerged as promising therapeutic agents for AD due to their neuroprotective effects, non-toxicity, multi-targeted mechanisms, and low cost (Zhang et al., 2014; Shang et al., 2020; Li et al., 2021). These macromolecules derived from diverse sources such as Chinese medicinal plants, marine organisms, plants, and fungi, target different pathological mechanisms of AD such as Aβ plaques, tau NFT, and neuroinflammation, Nrf2 activation and autophagy, as highlighted in Table 2 (Wang et al., 2018; Rathnayake et al., 2019; Li et al., 2021). The activation of the Nrf2 pathway enhances the expression of cytoprotective enzymes such as HO-1 (heme oxygenase-1) and NQO1 (NAD(P)H:quinone oxidoreductase 1) resulting in reduction of free radical formation and protection of neurons from oxidative damage, a mechanism that may also mitigate Aβ- and tau-associated neurotoxicity in AD (Cao et al., 2017). It was reported that *Lycium barbarum* polysaccharides (LBP) exhibit potential to reduce oxidative stress, apoptosis, and exert autophagy-modulating effects, reducing neuronal damage in AD models. In Aβ_1 − 42_-induced AD rats, *Lycium barbarum* polysaccharide (LBP) orally administered at 150–300 mg/kg/day for 4 weeks improved cognitive function, reduced neuroinflammation and AChE activity. Similarly, in a transient global ischemia rat model, oral LBP administered for 1 week before and after ischemia reduced CA1 (Cornu Ammonis 1) neuronal loss, lowered the damage score to 1.1 ± 0.1 and improved memory performance (Xing et al., 2016; Lu et al., 2025). Similarly, acemannan from *Aloe vera* also enhances antioxidant defenses and immune modulation, supporting cognitive function (Liu et al., 2019). Polysaccharides from fungi (e.g., lentinan), plants (e.g., *Astragalus membranaceus* polysaccharides), and marine algae (e.g., fucoidan) have been explored as therapeutic formulations. Lentinan, is an approved adjuvant for cancer therapy in some countries and has also demonstrated neuroprotective effects in preclinical models of AD. In contrast, fucoidan and *Astragalus* polysaccharides have primarily shown anti-AD effects and neuroprotection in preclinical studies, with only limited early-stage clinical investigations for neurodegenerative diseases such as AD, Parkinson's disease, and amyotrophic lateral sclerosis (Zhang et al., 2011; Zhang Z. et al., 2022). To further explain the therapeutic potential of polysaccharides, the following section explores the structure-activity relationships that govern their neuroprotective effects in AD, providing a foundation for optimizing their design and application. Figure 4 below shows a schematic illustration of sources and mechanisms of therapeutic effects of polysaccharides which are explained in more detail in the later sections.

## Structure-activity relationship (SAR) of polysaccharides in AD

The therapeutic efficacy of natural polysaccharides in AD is closely linked to their structural characteristics, including molecular weight (MW), monosaccharide composition, glycosidic linkages, and functional group modifications such as sulfation or methylation. It is necessary to understand the SAR for optimizing polysaccharide-based therapies for enhancing their neuroprotective effects, bioavailability, and clinical applicability.

### Bioactivity and molecular weight

The molecular weight of polysaccharides significantly affects their biological activity and bioavailability. Low-molecular-weight polysaccharides often demonstrate superior protective effects due to better cellular uptake and penetration of blood-brain barrier (BBB). A low-molecular-weight galactan isolated and purified from *Cantharellus cibarius* (named CCP, with molecular weight 5 kDa) was reported to reduce Aβ plaque deposition and neuroinflammation in APP/PS1 mice by activating the C3-centered complement system, outperforming its higher-molecular-weight counterparts (Yang et al., 2019; Zhou et al., 2023). Similarly, alginate oligosaccharides (AOS, < 10 kDa) derived from brown algae (*Laminaria hyperborea*, and *Undaria pinnatifida*) inhibit β-site amyloid precursor protein (APP) cleaving enzyme 1 (BACE1), a key aspartyl protease responsible for the β-secretase cleavage of APP leading to Aβ peptide production which is a hallmark of AD pathology. By inhibiting BACE1, AOS can reduce Aβ generation and also enhance autophagy in SH-SY5Y cells, exhibiting greater efficacy than higher-molecular-weight alginates due to improved solubility and cellular interactions (Bi et al., 2021). Recent studies further confirm that low-molecular-weight polysaccharides from *Astragalus membranaceus* (APS, ~10–50 kDa) effectively activate Nrf2 signaling and reduce Aβ accumulation in AD mouse models, due to their ability to cross cellular membranes (Qin et al., 2020; Tang et al., 2024). These findings suggest that reducing molecular weight through enzymatic hydrolysis or chemical depolymerization enhances polysaccharide efficacy in AD.

### Monosaccharide composition and glycosidic linkages

The monosaccharide composition and glycosidic linkages of polysaccharides influence their interactions with biological targets. Polysaccharides rich in galactose, mannose, or glucuronic acid exhibit potent anti-AD effects (Zhu et al., 2024). It has been reported that *Hericium coralloides* polysaccharide (HCP), composed of α-D-galactopyranose (Galp) units linked mainly via α1 → 6 bonds and substituted with α-L-fucopyranose (Fucp) at the O-2 position, enhances autophagic flux and reduces neuroinflammation in APP/PS1 mice (Guan et al., 2023). Intragastric administration of purified polysaccharides from fermented mycelium of *Hericium erinaceus* named PHEB in the APP/PS1 mice (25, and 100 mg/kg/day for 6 weeks) significantly improved memory performance (decreased Morris Water Maze (MWM) latency, increased step-down avoidance), reduced hippocampal Aβ_1 − 42_ (~31% reduction) and p-tau, and restored ACh, choline acetyltransferase (ChAT), and antioxidant enzyme levels (Hu et al., 2021b). The galactan backbone facilitates interactions with microglial receptors, promoting anti-inflammatory M2 phenotypes. Similarly, *Dendrobium officinale* polysaccharide (DOP), composed primarily of mannose and glucose with β-1,4-glycosidic linkages, reduces Aβ and tau accumulation in SAMP8 mice by regulating microglial polarization (Feng et al., 2019). Another study on *Durio zibethinus* seed polysaccharide (DSPP-1), rich in galactose and glucuronic acid, demonstrated reduced Aβ aggregation in *Caenorhabditis elegans* CL4176 models, attributed to its β-1,3/1,6-galactan structure that protects against free radicals damage (Xiao et al., 2023). These studies highlight that specific monosaccharide compositions, particularly galactose and mannose, are critical for targeting AD pathologies, while the influence of α- or β-glycosidic linkages on activity remains to be fully elucidated for AD. Galactose-rich polysaccharides are hypothesized to interact more effectively with lectin-type receptors expressed on immune cells. This potentially contributes to treating inflammation and help in immunomodulatory effects. However, direct receptor-binding evidence remains limited for most polysaccharides investigated in AD models. Glucuronic acid residues provide negative charge and improve solubility, which facilitates electrostatic interactions with positively charged domains of amyloid-β fibrils, thereby inhibiting their aggregation and reducing oxidative stress. Such structure-function relationships have been highlighted in recent studies of marine algal polysaccharides showing that acidic, galactose-containing polysaccharides exhibit potent protective effects for neurons through antioxidant, anti-amyloidogenic, and anti-inflammatory mechanisms (Naik et al., 2024).

Although several structure-function relationships have been proposed, direct mechanistic evidence remains limited for many polysaccharides. In several cases, associations between monosaccharide composition, charge distribution, or branching patterns and biological activity are inferred from molecular docking, computational modeling, or studies conducted in non-AD systems. For example, sulfated polysaccharides and acidic polysaccharides have been predicted to interact with amyloidogenic proteins through electrostatic interactions. Galactose containing structures may exhibit enhanced affinity for lectin-like receptors involved in immune regulation. However, quantitative receptor-binding kinetics and direct structural validation remain limited. Future studies combining molecular docking, molecular dynamics simulations, surface plasmon resonance, isothermal titration calorimetry, and receptor-blocking experiments will be important for establishing causal SAR relationships and validating proposed mechanisms.

## Modification of functional groups

Different chemical modifications, such as sulfation, methylation, or selenization, enhance polysaccharide bioactivity by altering their charge, solubility, and receptor-binding affinity. Sulfated polysaccharides from marine algae, such as fucoidan from *Undaria pinnatifida* and alginate-derived mannuronate oligosaccharides (MOS) have demonstrated efficiency in anti-Aβ and anti-inflammatory effects. It has been reported that sulfate groups increase negative charge, facilitating interactions with positively charged amyloid fibrils, thus inhibiting aggregation (Bi et al., 2021; Tang et al., 2024; Wako et al., 2024). A study on sulfated polysaccharides from *Ulva lactuca* demonstrated increased cholinesterase inhibition and reduced neuroinflammation in HT-22 cells, attributed to the ability of sulfate groups to modulate acetylcholinesterase activity (Olasehinde et al., 2019b). Methylated polysaccharides, such as the 3-O-methylated galactan from *Cantharellus cibarius* (CCP) has shown improved macrophage activation and Aβ clearance, likely due to increased hydrophobicity and receptor specificity (Yang et al., 2019). Polysaccharides from *Acanthopanax senticosus* have been reported for strong free radical scavenging activity. A purified polysaccharide fraction name CQ-1, extracted from *Acanthopanax senticosus*, increased the activities of superoxide dismutase (SOD) and catalase (CAT) and reduced the malondialdehyde (MDA) and reactive oxygen species (ROS) levels in *Caenorhabditis elegans*. This resulted in improving oxidative stress resistance and lifespan (Bai et al., 2025). Moreover, chemical modification by selenization has been reported to enhance the antioxidant capacity of *A. senticosus* polysaccharides compared with unmodified polysaccharides (Li X. et al., 2024). However, to the best of our knowledge, the effect of acetylation on the antioxidant or neuroprotective activity of these polysaccharides has not yet been investigated. These modifications underscore the potential of improved functionalization to optimize polysaccharide efficacy.

The inconsistency across different studies remains a major challenge despite encouraging findings. The presence of variations across different polysaccharides in terms of purity, extraction methods, structural characterizations, and dosing regimens prevent direct comparisons of outcomes. The effects on experimental models also vary where most of the evidence is drawn from *in vitro* and animal model studies, while human clinical data is limited. These challenges highlight the need for standardized extraction protocols, structural analysis, dose-optimization protocols, and roadmap for preclinical to clinical translational studies for validating therapeutic efficiency (Du et al., 2024; Luo et al., 2024).

Although molecular weight and monosaccharide composition influence polysaccharide bioactivity but higher-order structural features also influence this characteristic. Structural variations in synthetic heparan sulfate oligosaccharides (particularly chain length and sulfation patterns) significantly modulate binding affinity to amyloid-β and reduce Aβ-induced cellular toxicity. In dietary polysaccharides having immunomodulatory activity, branching degree and conformational flexibility can alter solubility, receptor accessibility, and binding to immune cell receptors such as TLR4 and Dectin-1. Triple-helix conformations, reported for several bioactive polysaccharides, may enhance receptor recognition and immunomodulatory activity compared to random-coil structures (Wang et al., 2021; Chen et al., 2022). Charge density is also critical, especially for sulfated polysaccharides, because sulfate groups mediate electrostatic interactions with amyloidogenic proteins and cell-surface receptors. Importantly, activity may depend not only on total sulfation but also on sulfation position and chain length. Heparin and heparan-sulfate studies show that defined sulfation motifs influence Aβ binding and aggregation behavior (Wang et al., 2021; Zhou et al., 2022). Similarly, acetylation patterns may modify hydrophobicity, chain conformation, and receptor affinity, although evidence specific to AD remains limited. Molecular docking and modeling studies provide useful insights into polysaccharide-Aβ and polysaccharide-receptor interactions. But these approaches require validation through binding kinetics, surface plasmon resonance, isothermal titration calorimetry, and cell-based receptor-blocking assays before concrete SAR conclusions can be derived.

Table 3A shows a summary of key structural features of polysaccharides and their corresponding anti-AD effects, highlighting the role of molecular weight, monosaccharide composition, glycosidic linkages, and functional modifications, Table 3B shows the current level of mechanistic evidence proposed for polysaccharide use against AD.

**Table 3A:** Structure-activity relationships of polysaccharides in Alzheimer's disease.

Polysaccharide source	Structural feature	Reported neuroprotective effect in AD models	References
*Cantharellus cibarius* polysaccharides (CCP)	Low-molecular-weight (5 kDa), 3-O-methylated α-1,6-galactan	Reduces Aβ plaques, enhances macrophage activation, suppresses neuroinflammation	Yang et al., 2019; Zhou et al., 2023
*Hericium coralloides* polysaccharides (HCP)	α-(1 → 6)-galactan backbone with α-L-fucose substitutions at O-2	Enhances autophagic flux, reduces Aβ plaques, improves cognitive function	Guan et al., 2023
*Dendrobium officinale* polysaccharides (DOP)	Mannose/glucose-rich polysaccharide with β-(1 → 4)-glycosidic linkages	Modulates microglial polarization, reduces Aβ and tau accumulation	Feng et al., 2019
*Durio zibethinus* polysaccharides (DSPP-1)	Galactose/glucuronic acid-rich β-(1 → 3)/(1 → 6)-galactan	Reduces Aβ aggregation, and attenuates oxidative stress	Xiao et al., 2023
*Ulva lactuca* polysaccharides	Sulfated polysaccharides	Suppresses cholinesterase activity and neuroinflammation	Olasehinde et al., 2019b
*Laminaria hyperborea* oligosaccharide (AOS)	Low-molecular-weight alginate oligosaccharides	Inhibits BACE1, enhances autophagy, reduces Aβ levels	Bi et al., 2021
*Acanthopanax senticosus* polysaccharides	Acetylated polysaccharides	Attenuates oxidative stress and improves antioxidant defenses	Wang et al., 2022

Aβ, amyloid beta; CCP, *Cantharellus cibariu*s polysaccharide; HCP, *Hericium coralloides* polysaccharide; DOP, *Dendrobium officinale* polysaccharide; DSPP-1, *Durio zibethinus* seed polysaccharide fraction 1; AOS, alginate oligosaccharides; BACE1, β-site amyloid precursor protein cleaving enzyme 1.

**Table 3B:** Proposed structure and function relationships and current level of mechanistic evidence for polysaccharides in AD.

Structural feature	Proposed mechanism	Evidence type	Evidence strength
Low molecular weight	Improved uptake/bioavailability	Animal + *in vitro*	Moderate
Galactose-rich composition	Lectin receptor interaction, immune modulation	Indirect studies, docking, immunology	Preliminary
Sulfation	Electrostatic interaction with Aβ	*In vitro* + modeling	Moderate
Triple-helix conformation	Enhanced receptor recognition	Non-AD polysaccharide studies	Preliminary
High charge density	Protein binding and anti-aggregation effects	*In vitro*	Preliminary
Acetylation	Conformational changes, receptor affinity	Limited studies	Preliminary

**Figure 5:**
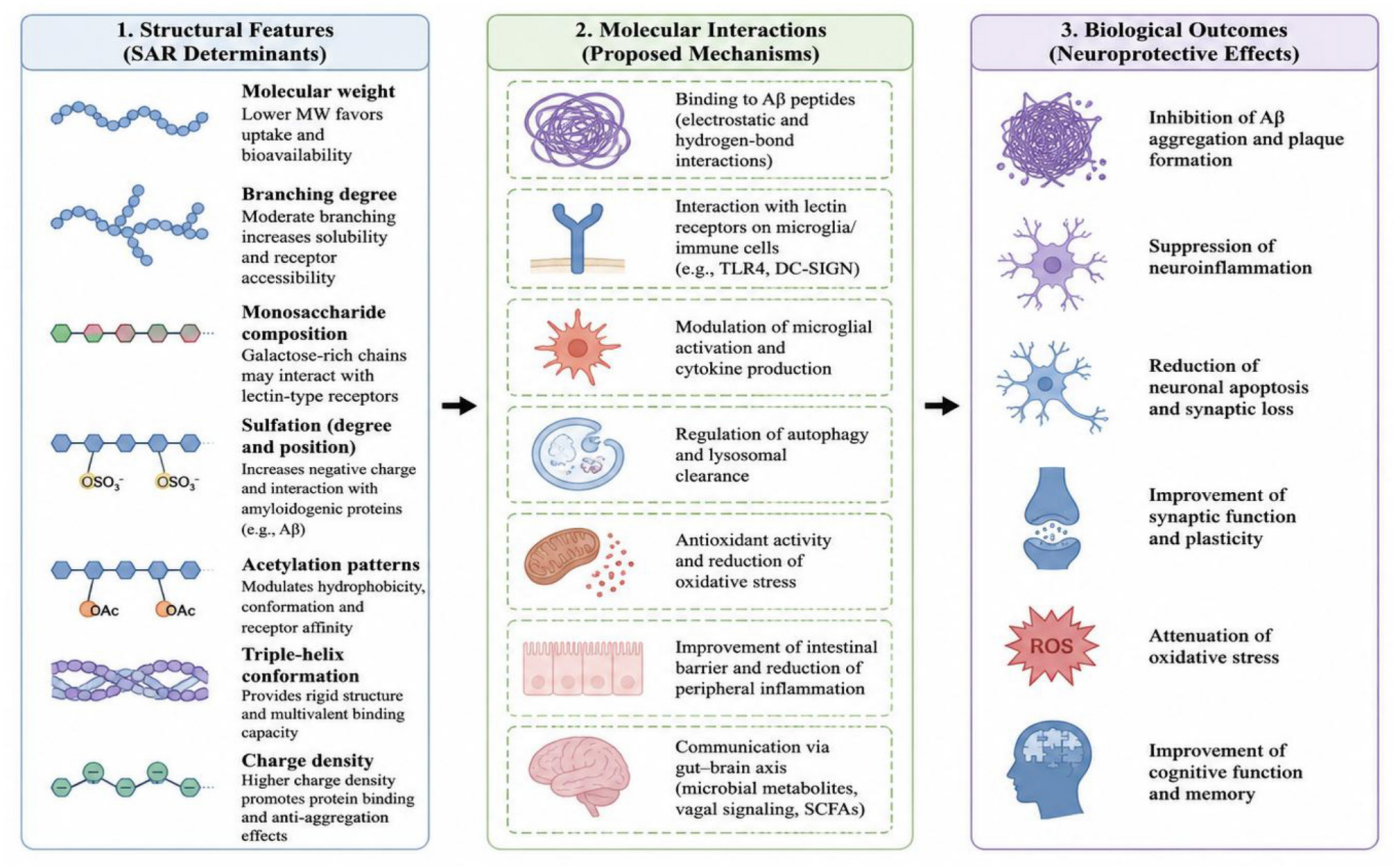
Structural determinants influencing polysaccharide neuroprotective activity in Alzheimer's disease (AD). MW, molecular weight; Aß, amyloid-ß; TLR4, toll-like receptor 4; DC-SIGN, dendritic cell-specific intercellular adhesion molecule-3-grabbing non-integrin; SCFAs, short-chain fatty acids; ROS, reactive oxygen species; SAR, structure-activity relationship.

The major structural determinants proposed to influence bioactivity of polysaccharides in AD along with their putative molecular interactions and downstream neuroprotective effects, are summarized in Figure 5.

### Challenges and future directions

Although structural features significantly influence polysaccharide activity, challenges remain in standardizing their composition and improving bioavailability. The variations in monosaccharide composition and linkage patterns across different sources complicate the reproducibility. Moreover, high-molecular-weight polysaccharides often face poor BBB penetration. Recently, nanocarriers containing curcumin were coated with modified red blood cell membranes to treat AD. Nanoparticles could be recognized by receptors on the BBB, and deliver drugs to the brain causing a delay in the progression of AD (Gu et al., 2024). Similar strategies could be adapted for polysaccharides, where conjugation with nanoparticles or coating with biomimetic membranes may enhance BBB penetration and prolong circulation, though direct experimental evidence in polysaccharide systems remains limited. Detailed strategies for structural modifications have been discussed in the SAR section and Table 3A. The key challenge is translating these mechanistic insights into clinically viable formulations that are discussed in this section further.

Despite reports about *in vitro, in vivo* studies, most polysaccharides show poor oral bioavailability, and limited ability to cross the blood-brain barrier (BBB), due to their high molecular weight and hydrophobicity (Yang et al., 2023; Huang et al., 2024). Transmembrane diffusion (a primary mechanism for most drugs), is favored by low molecular weight and high lipid solubility. Although no absolute molecular-weight cutoff exists, the influence of size is inversely related to the square root of molecular weight, and some compounds larger than 600 Da cross the BBB sufficiently using this mechanism (Banks, 2009; Pardridge, 2020). Intravenous administration of *Angelica sinensis* polysaccharide results in rapid ASGPR-mediated uptake into liver parenchymal cells. This causes increased hepatic accumulation that reduces plasma exposure and shortens circulation half-life (Zhang et al., 2018). Interactions with lectins, serum proteins, and other glycan-binding molecules influence clearance, cellular uptake, immunological responses, and biological activity (Varki, 2017). Current literature similarly emphasize that poor oral bioavailability, rapid metabolism, limited blood-brain barrier penetration, and insufficient clinical validation remain the principal hurdles which prevent the successful translation of natural neuroprotective compounds into clinical practice (Sarkar et al., 2025; Datta et al., 2026). Therefore, most of the reported neuroprotective effects may arise through indirect mechanisms involving modulation of systemic inflammation, peripheral immunity, or the gut-brain axis instead of substantial accumulation within the central nervous system. Future studies should therefore incorporate comprehensive pharmacokinetic analyses such as oral bioavailability, plasma half-life, metabolic fate, brain-to-plasma concentration ratios, and biodistribution profiling, to establish whether observed therapeutic effects result from direct brain exposure or peripheral mechanisms.

Studies on the structure-activity relationships of polysaccharides are limited due to low purity and high heterogeneity. Variability in molecular weight, monosaccharide composition, sulfation, acetylation, glycosidic bonds, chain conformation, and branching degree further complicates identification of reproducible active components (Chen et al., 2022). In addition, many studies lack comprehensive brain pharmacokinetic profiling (Pardridge, 2020). Most efficacy data derive from first-generation transgenic APP/PS1 mouse models, which reproduce familial amyloid pathology through FAD mutations but fail to replicate sporadic late-onset AD or extensive neuronal loss (Drummond and Wisniewski, 2017; Sasaguri et al., 2017). Notably, numerous agents that demonstrated efficacy in preclinical AD models have subsequently failed in clinical trials, highlighting the challenges of translating experimental findings into effective human therapies (Mullane and Williams, 2013). For polysaccharides specifically, mechanistic uncertainty remains an additional barrier, as reported protection of neuronal damage may arise through direct neuronal actions, modulation of neuroinflammation, gut microbiota-derived metabolites, or systemic immune regulation. Future translational efforts should therefore prioritize chemically defined polysaccharide fractions, GMP-compatible manufacturing, rigorous pharmacokinetic and pharmacodynamic characterization, validated biomarkers, and well-designed multicentre clinical trials to establish efficacy and safety in human AD populations.

Advanced strategies such as enzymatic depolymerization, sulfation or methylation, nanoparticle encapsulation, and conjugation with BBB-penetrating ligands are being explored to enhance brain delivery and pharmacokinetic performance (Arlov et al., 2021; Cheng et al., 2023; De Capua et al., 2024; Han et al., 2025). Future studies should integrate systematic pharmacokinetic profiling with well-designed clinical trials to establish optimal dosing, safety, and therapeutic efficacy in humans (Cummings et al., 2025). Overall, a concise research map should include; (i) Standardization of extraction and characterization; development of good manufacturing practice (GMP) compatible standard operating procedures (SOPs); reporting monosaccharide composition, molecular weight (MW) distribution (e.g., size-exclusion chromatography with multi-angle light scattering), glycosidic linkages (nuclear magnetic resonance), sulfate and acetyl content, endotoxin levels, bioburden, and batch-to-batch specifications. (ii) Inter-lab reproducibility by creating reference materials and assays to harmonize potency readouts (e.g., anti-Aβ aggregation, microglial modulation). (iii) Pharmacokinetics and pharmacodynamics (PK/PD) and blood–brain barrier (BBB) by conducting oral and intravenous pharmacokinetic studies with brain/plasma ratios, metabolite identification, and population pharmacokinetic modeling; integration with pharmacodynamic biomarkers such as Nrf2 targets and cytokines. (iv) Formulation and delivery through head-to-head comparisons of native vs. depolymerized/sulfated derivatives and nano-enabled formats with predefined release criteria and stability. (v) Translational preclinical package through the use of AD transgenic models with randomization/blinding, sample-size justification, and toxicity/immunogenicity screens, and (vi) Clinical pathway beginning with Phase 1 single ascending dose (SAD) and multiple ascending dose (MAD) studies for safety and pharmacokinetics (PK), followed by adaptive multicenter Phase 2 randomized controlled trials (RCTs) including biomarker panels [cerebrospinal fluid (CSF) Aβ42/40, phosphorylated tau (p-tau), neurofilament light chain (NfL)] and cognitive outcomes along with integration of chemistry, manufacturing, and controls (CMC) scale-up and quality control.

Polymeric nanoparticles have been explored as nanoencapsulation systems to improve therapeutic stability, prolong circulation through PEGylation, enhance cellular uptake, and facilitate blood-brain barrier transport in stroke, AD and PD models. Exosome-based systems have similarly been used to load therapeutics for better stability and brain delivery, cellular uptake, and crossing of blood-brain barrier that prevents damage to neurons in models of AD and PD stroke, and multiple sclerosis (Saraiva et al., 2016; Vahab et al., 2025). Intranasal administration has also attracted considerable interest because it may enable direct nose to brain transport while partially bypassing the blood-brain barrier and first-pass metabolism (Lochhead and Thorne, 2012). Although these technologies have demonstrated encouraging results in experimental neurodegenerative disease models, their application to polysaccharide-based therapies for AD is still at an early stage. Future studies should evaluate the safety, biodistribution, scalability, and long-term efficacy of such delivery platforms to determine whether they can significantly improve the translational potential of neuroprotective polysaccharides.

Regulatory and safety aspects also remain underexplored. Variability in extraction and purification challenges reproducibility, and successful translation will require good manufacturing practice (GMP) compliance, rigorous quality control, and systematic testing for toxicity and immunogenicity. These steps are critical for eventual clinical approval (Du et al., 2024; Luo et al., 2024).

According to the 2024 Alzheimer's Association revised criteria for diagnosis and staging of Alzheimer's disease (Jack et al., 2024), Core 1 biomarkers like amyloid PET, approved CSF ratios (for example, p-tau181/Aβ42, t-tau/Aβ42, or Aβ42/40), and accurate plasma phosphorylated-tau assays (particularly p-tau217) are sufficient to establish a diagnosis of AD. Core 2 biomarkers (tau PET and selected fluid tau analytes such as MTBR-tau243) and non-specific biomarkers (NfL and GFAP) are used, in conjunction with Core 1 results, for biological staging of disease severity, to provide prognostic information, and as indicators of biological treatment effects. Abnormal Core 2 results further increase confidence that AD is contributing to clinical symptoms (Palmqvist et al., 2020; Jack et al., 2024). Therefore, future investigations should determine whether polysaccharide-mediated reductions in amyloid deposition, tau pathology, neuroinflammation, and neurodegeneration are accompanied by measurable changes in these clinically relevant biomarkers. The establishment of such links would substantially strengthen translational relevance and facilitate comparison with emerging disease-modifying therapies currently under clinical evaluation.

Fucoidan is a sulfated polysaccharide from brown macroalgae which exhibits antithrombotic and thrombolytic properties such as through inhibition of the tPA-PAI1 complex. It also binds P-selectin on platelets which enables its use for thrombus imaging. Clinical safety of radiolabelled fucoidan has been demonstrated in a Phase I trial, with additional safety data available from multiple clinical studies of fucoidan extracts in other indications (Fitton et al., 2019). Future preclinical and clinical investigations should therefore incorporate comprehensive toxicological assessments, immunogenicity testing, complement activation analyses, and long-term safety monitoring to establish the risk-benefit profile of polysaccharide-based interventions for AD.

Large-scale proteomic analyses of AD brain have identified protein coexpression modules enriched in energy metabolism and glial markers. This correlates strongly with neuropathology, cognitive decline, and early changes observed in asymptomatic stages. Integrative multi-omics approaches using combination of transcriptomic and proteomic data have further revealed immune signaling and metabolic pathways associated with disease risk and progression (Johnson et al., 2020; Venkatesh et al., 2025). Such approaches could help clarify how structurally distinct polysaccharides influence cellular signaling networks, host-microbiome interactions, and downstream biological responses. Glycomics may particularly provide valuable insights into polysaccharide-protein interactions and glycan-mediated signaling events that remain poorly understood. Future studies integrating multi-omics datasets with pharmacokinetic, biomarker, and behavioral outcomes may improve mechanistic resolution and facilitate translation toward clinically relevant therapeutic strategies.

The 2026 AD drug development pipeline includes 158 drugs being evaluated across 192 active clinical trials, reflecting continued expansion and diversification of the therapeutic landscape. Disease-targeted therapies account for approximately 73% of the pipeline, while repurposed agents represent 35% of drugs under investigation. Collectively, these trials require more than 54,000 participants, highlighting the substantial global effort directed toward development of disease-modifying treatments for AD (Cummings et al., 2026). Sodium oligomannate (GV-971) is an oligosaccharide derived from marine brown algae with a novel proposed mechanism of action, and first Phase-3 clinical trial had been completed in China (Xiao et al., 2021). However, none of these candidates are polysaccharide-based, which highlights the absence of clinical translation for these natural compounds and the need for well-designed human trials.

In line with the Methods, the findings form different studies are presented below according to model type (*in vitro*, animal, clinical), with sample sizes noted where available and evidence tiers (strong, moderate, preliminary) applied to indicate robustness. This approach enables clearer distinction between preliminary observations and more substantiated evidence.

## Fungal polysaccharides and AD

Fungal polysaccharides have shown significant promise as multi-targeted, low-toxicity therapeutics for AD. The following section has been arranged to show therapeutic potential of fungal polysaccharides based on pathological processes. Evidence for fungal polysaccharides is moderate overall as multiple *in vivo* AD model studies report cognitive and biomarker improvements, though sample sizes are typically modest and blinding/randomization details are often not reported (indicated with NR where absent).

## Polysaccharides targeting neuroinflammation and oxidative stress

Several fungal polysaccharides exert strong anti-inflammatory and antioxidant effects crucial for treating progressive AD. Polysaccharides from species like *Inonotus obliquus, Codonopsis pilosula, Sparassis crispa*, and *Amanita caesarea* exhibit neuroprotective effects. *C. pilosula* polysaccharides protected Aβ1–40-induced PC12 cell models in *in vitro* study at concentrations of 25–300 μg/ml for 24 h and resulted in improved mitochondrial energy metabolism and reduced stress caused by free radicals through CD38/NAD^+^ signaling. Model type: *in vitro* (PC12 neuronal cells). Sample size: cell replicates as reported. Evidence tier: preliminary (single *in vitro* study without *in vivo* validation). Similarly, *A. caesarea* polysaccharides was reported to demonstrate anti-AD activity in APP/PS1 transgenic mice in *in vivo* study, where their administration improved cognitive function and cholinergic system activity, while alleviating inflammation through activation of the Nrf2 signaling pathway (Hu et al., 2021a,c; Tang et al., 2024). Model type: *in vivo* (APP/PS1 mice), oral 2.5 or 5 mg/kg for 6 weeks. Sample size: *n* = 9 (behavioral tests), *n* = 3 (IHC), *n* = 6 (ELISA/biochemical assays). Evidence tier: moderate (single-lab *in vivo* study with behavioral and biomarker outcomes) (Hu et al., 2021a). *Cantharellus cibarius* polysaccharides alleviate AD-like symptoms in mice by inhibiting neuroinflammation and oxidative stress. Model type: *in vivo* (APP/PS1 mice), oral 50 mg/kg for 8 weeks. Sample size: *n* = 10 per group. Evidence tier: moderate (single-lab *in vivo* study with behavioral, biochemical, and histological endpoints) (Zhou et al., 2023). *Hericium erinaceus* polysaccharides mitigate oxidative stress by regulating calcium homeostasis, offering neuroprotection in AD models. Model type: *in vivo* (APP/PS1 transgenic mouse model), oral 25–100 mg/kg for 6 weeks. Sample size: *n* = 10 per group. Evidence tier: moderate (based on a single *in vivo* model but lacking clinical data) (Hu et al., 2021b).

## Polysaccharides regulating amyloid pathology and APP expression

Some fungal polysaccharides directly influence amyloid pathology and APP metabolism. *Hericium coralloides*, an edible fungus of the Hericiaceae family, is a notable source of polysaccharides useful against neuronal damage (Cummings et al., 2023; Williams et al., 2023). Its fruiting bodies reduce Aβ plaque formation in APP/PS1 mouse models, preventing damage caused by free radicals and improving cognitive function. Model type: *in vivo* (APP/PS1 transgenic mice), oral administration (dose: 500 mg/kg HC dissolved in 0.2% CMC-Na, 5 mL/kg). Sample size: *n* = 8 per group. Evidence tier: moderate (single *in vivo* study demonstrating behavioral and histological improvements) (Guan et al., 2023). Similarly, alcoholic extract of *Hericium erinaceus* mycelia, administered orally at low doses (3.2 mg/30 g body weight/day) and high doses (6.4 mg/30 g body weight/day) for 3 months, activates SIRT-1/ERK-1/2-mediated autophagy, upregulates LC3-II expression, and promotes the clearance of Aβ and α-synuclein aggregates in A53T transgenic mice, thereby preserving dopaminergic neurons and improving motor function. Model type: *in vivo* (A53T transgenic mice), oral extract 3.2–6.4 mg/30 g/day for 3 months on alternate days. Sample size: *n* = 5 per group. Evidence tier: moderate (single-lab *in vivo* study, extract-based rather than purified polysaccharide) (Lin et al., 2023). Similarly, *Pleurotus eryngii* polysaccharides reduce APP production in aging rats and protect against Aβ-induced neurotoxicity in rat pheochromocytoma cells. Model types: *in vivo* (aging rats) and *in vitro* (PC12 cells). Dose: 100–200 mg/kg oral in rats. Sample size: *n* = 8–10 per group. Evidence tier: *moderate* (dual-model evidence, but limited to single-lab preclinical data) (Zhang C.-J. et al., 2020).

## Role of low-molecular-weight polysaccharides in enhancing bioavailability

Limitations associated with poor bioavailability of high-molecular weight polysaccharides have been investigated using low-molecular derivatives. As described above, a 5 kDa galactan (CCP) from *C. cibarius*, derived from a 20 kDa α-1,6-galactan partially methylated at O-3, activates macrophages and reduces Aβ plaque deposition in APP/PS1 mice via the C3-centered complement system. This improves memory and learning while suppressing neuroinflammation. This evidence is consistent with the previously described *in vivo* findings for *C. cibarius* polysaccharides, supporting a moderate evidence tier (Yang et al., 2019; Zhou et al., 2023). Such modifications are important for optimization of molecular size to achieve better therapeutic efficiency.

The combination of multiple advantages, such as broad-spectrum biological activities, minimal toxicity, and therapeutic potential makes fungal polysaccharides as promising agents for AD management. Further clinical and preclinical studies are required to translate these findings into clinical applications.

## Polysaccharides from algae and AD

Literature shows that evidence for algal polysaccharides is preliminary to moderate as most findings derive from *in vitro* neuronal models, with a few *in vivo* animal studies, while clinical data are still lacking. Sulfated polysaccharides from marine algae, rich in monosaccharides and sulfate groups, exhibit diverse biological activities, including prevention of oxidative stress, inflammation, and immunomodulatory effects relevant to AD (Mayakrishnan et al., 2013; Ngo and Kim, 2013). Fucoidans have demonstrated protective effects in *in vivo* model transgenic *Caenorhabditis elegans* (Wang et al., 2018) and in d-galactose-induced cognitive dysfunction in mice (Wei et al., 2017). They have also attenuated Aβ25–35- or d-galactose-induced neurotoxicity in PC12 cells (Wei et al., 2017), and mitigated and neuronal damage along with damage caused by free radicals in HT-22 cells (Olasehinde et al., 2020) both representing *in vitro* models. However, their neuroprotective potential in AD remains underexplored. Model types: *in vivo* (*C. elegans*, d-galactose-induced mice) and *in vitro* (PC12, HT-22 cells). Sample sizes: *n* = 10 per group (mice); cell replicates as reported. Evidence tier: preliminary (mixed *in vitro* + small-animal studies; no AD transgenic mouse validation).

## Polysaccharides targeting oxidative stress and neuroinflammation

Sulfated polysaccharides from green algae (*Ulva lactuca, Ulva rigida*), red algae (*Gracilaria gracilis, Gelidium pristoides*), and brown algae (*Ecklonia maxima*) inhibit neuronal apoptosis and enhance cholinergic function in Zn-induced neurodegeneration in HT-22 hippocampal neuronal cells (Olasehinde et al., 2020). The therapeutic effects exhibited by these compounds may alleviate neuronal damage by AD. Model type: *in vitro* (HT-22 hippocampal neurons, Zn-induced damage). Sample size: cellular replicates as reported. Evidence tier: preliminary (single *in vitro* model; no animal or clinical validation).

## Alginate-derived oligosaccharides with multi-target effects

Brown seaweeds, including *Laminaria hyperborea, Undaria pinnatifida, Ascophyllum nodosum*, and *Macrocystis pyrifera*, are rich sources of alginate, an acidic polysaccharide composed of β-D-mannuronate and its C-5 epimer α-L-guluronate linked by 1,4-glycosidic bonds (Arne et al., 1967; Park et al., 2009). Alginate is processed via enzymatic digestion, oxidative-reductive depolymerization, or acid hydrolysis to yield alginate oligosaccharides (AOS), including poly-guluronate (PG) and poly-mannuronate (PM) derivatives (Xu et al., 2014b, 2016).

## Biological activities of alginate oligosaccharides

Alginate Oligosaccharides (AOS) exhibit a variety of useful biological activities such as neuroprotection (Zhou et al., 2015b; Bi et al., 2018b, 2019), immunomodulatory (Xu et al., 2014a, 2015; Fang et al., 2017), antioxidant (Tusi et al., 2011), and anti-inflammatory (Zhou et al., 2015a; Bi et al., 2018a) activities. In RAW264.7 macrophages, alginate oligosaccharides, at 0.1–1 mg/ml concentration for 24 h dose-dependently increased NO, ROS, and TNF-α production via NF-κB/MAPK signaling, confirming immunomodulatory and potential prevention against neuronal damage (Xu et al., 2014b). Model type: *in vitro* (RAW264.7 macrophages). Sample size: cell replicates. Evidence tier: preliminary (mechanistic immunomodulation *in vitro*; no neuronal or *in vivo* validation yet). The diverse and multidimensional biological activities of AOS make them promising future therapeutic agent for the treatment of AD.

## Mannuronate oligosaccharide (MOS) in amyloid modulation and autophagy

A recent study investigated an unsaturated MOS derived from PM using alginate lyase, and its structure was characterized by electrospray ionization mass spectrometry (ESI-MS). In AD cell models (N2a-sw cells, a neuroblastoma cell line expressing Swedish mutant APP, and primary cortex neurons from 3 × Tg-AD mice, a triple-transgenic AD model), treatment with 1 mg/ml MOS for 24 h reduced Aβ levels by inhibiting BACE1, prevented Aβ oligomer aggregation, and enhanced autophagy; therefore, promoting clearance of Aβ and APP (Bi et al., 2021). Model types: *in vitro* (N2a-sw neuroblastoma cells, primary neurons from 3 × Tg-AD mice). Sample size: cell replicates. Evidence tier: preliminary (strong mechanistic evidence *in vitro* but no *in vivo* confirmation in AD mouse models). The potential of MOS as a functional food ingredient underscores the value of algal polysaccharides in AD management, with a need for further validation in AD-specific models (e.g., APP/PS1 mice). The details of studies conducted on fungal and algal polysaccharides, are summarized in Table 4.

**Table 4:** Polysaccharides from Fungi and Algae and reported therapeutic details.

Polysaccharide source	Reported neuroprotective effect in AD models	Principal mechanism or study focus	References
*Inonotus obliquus*	Attenuates AD-related pathology	Modulation of Aβ burden and neuroinflammation	Tang et al., 2024
*Sparassis crispa*	Attenuates AD-related pathology	Modulation of Aβ burden and neuroinflammation	Tang et al., 2024
*Amanita caesarea*	Suppresses neuroinflammation	Activation of Nrf2 signaling pathway	Hu et al., 2021a
*Cantharellus cibarius* (CCP, 5 kDa)	Alleviates AD-like symptoms, reduces Aβ plaques, improves memory and learning	Low-molecular-weight α-(1 → 6)-galactan (5 kDa); modulation of neuroinflammation through the C3 complement pathway; structural characterization of CCP	Yang et al., 2019; Zhou et al., 2023
*Hericium coralloides*	Reduces Aβ pathology, improves cognitive performance, and attenuates oxidative stress	Structural characterization of HCP; enhancement of autophagic flux and suppression of neuroinflammation	Guan et al., 2023
*Pleurotus eryngii*	Reduces amyloid precursor protein (APP) expression	Regulation of APP production in AD models	Zhang C.-J. et al., 2020
*Hericium erinaceus*	Attenuates oxidative stress	Regulation of calcium homeostasis	Hu et al., 2021b
*Ulva lactuca*	Inhibits neuronal apoptosis and improves cholinergic function	Acetylcholinesterase inhibition and suppression of neuroinflammation	Olasehinde et al., 2020
*Ulva rigida*	Inhibits neuronal apoptosis and improves cholinergic function	Acetylcholinesterase inhibition and suppression of neuroinflammation	Olasehinde et al., 2020
*Gracilaria gracilis*	Inhibits neuronal apoptosis and improves cholinergic function	Acetylcholinesterase inhibition and suppression of neuroinflammation	Olasehinde et al., 2020
*Gelidium pristoides*	Inhibits neuronal apoptosis and improves cholinergic function	Acetylcholinesterase inhibition and suppression of neuroinflammation	Olasehinde et al., 2020
*Ecklonia maxima*	Inhibits neuronal apoptosis and improves cholinergic function	Acetylcholinesterase inhibition and suppression of neuroinflammation	Olasehinde et al., 2020
*Laminaria hyperborea*	Reduces Aβ levels, enhances autophagy	BACE1 inhibition and prevention of Aβ oligomer aggregation	Bi et al., 2021
*Undaria pinnatifida*	Reduces Aβ levels, enhances autophagy	BACE1 inhibition and prevention of Aβ oligomer aggregation	Bi et al., 2021
*Ascophyllum nodosum*	Reduces Aβ levels, enhances autophagy	BACE1 inhibition and prevention of Aβ oligomer aggregation	Bi et al., 2021
*Macrocystis pyrifera*	Reduces Aβ levels, enhances autophagy	BACE1 inhibition and prevention of Aβ oligomer aggregation	Bi et al., 2021

Aβ, amyloid beta; APP, amyloid precursor protein; HCP, *Hericium coralloides* polysaccharide; CCP, *Cantharellus cibarius* polysaccharide; BACE1, β-site amyloid precursor protein cleaving enzyme 1.

This table is only a descriptive supplementary classification and detailed model types and evidence appraisal are already summarized in Table 2.

## Plant polysaccharides and AD

Plants have long been a source of bioactive compounds for treating NDDs, and plant polysaccharides have emerged as a key biomacromolecule due to their multi-targeted therapeutic effects (Park et al., 2017; Kakar et al., 2020a,b, 2022; Xiao et al., 2024; Lu et al., 2025). Various studies have been conducted to establish the therapeutic efficiency of polysaccharides for treating AD. Evidence for plant-derived polysaccharides is moderate overall. Several rodent studies report behavioral/biochemical benefits, alongside numerous cell-based findings; clinical data are currently lacking, and sample sizes are often modest which are mentioned wherever available.

## Polysaccharides targeting neuroinflammation and microglial modulation

Several plant polysaccharides exhibit strong anti-neuroinflammatory effects by modulating microglial activity. Traditional Chinese Medicine (TCM) extensively reports the neuroprotective potential of *Dendrobium* species (Orchidaceae), the epiphytic herbs used as food and medicine (Lam et al., 2015). Although *Dendrobium* polysaccharides are known for immunomodulatory effects (Zha et al., 2014; Xie et al., 2016), their role in AD is underexplored. A study investigated *Dendrobium officinale* polysaccharides (DOP) in BV2 microglial cells and senescence-accelerated mouse prone 8 (SAMP8) models. DOP promoted a microglial phenotype switch from pro-inflammatory M1 to anti-inflammatory M2, reduced Aβ and phosphorylated tau accumulation, and enhanced Aβ degradation, attenuating cognitive impairment in SAMP8 mice. DOP was supplemented in drinking water (0.3% to 0.6%), to mice 4–7 months of age, and it reduced hippocampal Aβ_42_ and p-tau, promoted M1 → M2 microglial switching, and improved learning and memory in fear conditioning and MWM test. Model types: *in vitro* (BV2 microglia) and *in vivo* (SAMP8 and SAMR1 mice); dose: 0.02% (w/v) DOP in drinking water, equivalent to 40 mg/kg/day (for 3 months, 4–7 months of age). Sample size: *n* = 12 per group. Evidence tier: *moderate* (single-lab *in vivo* + mechanistic microglial data) (Feng et al., 2019).

Kunlun Chrysanthemum (*Coreopsis tinctoria*), a North American native plant cultivated in China's Xinjiang province, contains polysaccharides and other bioactive compounds, such as flavonoids and phenolic acids, with anti-neuroinflammatory and antioxidant potential (Dias et al., 2010; Guo et al., 2015; Li et al., 2015; Wang T. et al., 2015; Wang W. et al., 2015). Three oligosaccharides, CT70-1A (1221 Da), CT70-1B (1318 Da), and CT70-2 (1247 Da) were isolated from its flowers, and characterized as complex glucans, galactans, and mannans terminated by α-D-Glcp (α-D-glucopyranose) and (α-L-Araf-L-arabinofuranose) residues (Cox, 1982b; Yu et al., 2021). In N9 microglial cells, these oligosaccharides inhibited nitric oxide (NO) production (IC_50_: 0.23–0.27 mM), indicating anti-neuroinflammatory activity, consistent with flavonoid-mediated NO inhibition. Model type: *in vitro* (N9 microglial cells). Sample size: cell replicates as reported. Evidence tier: preliminary (microglial NO inhibition without *in vivo* AD validation) (Cox, 1982a; Li et al., 2023b).

## Polysaccharides attenuating oxidative stress and apoptosis

Some plant polysaccharides exert potent effects against oxidative stress and protect neurons from apoptosis induced by this stress. *Annona muricata*, a tropical plant used in TCM, yields leaf polysaccharides (ALPs) with potent scavenging of free radicals and neuroprotection relevant to AD. In H_2_O_2_-treated HT22 hippocampal neuronal cells, ALPs (31–250 μg/ml, 2 h pretreatment) enhanced cell survival, reduced ROS, Ca^2+^ accumulation, and MDA levels, and restored the expression of SOD. ALPs also modulated key cell signaling mediators such as cytochrome c, Bax, MAPKs (ERK, JNK, p38), NF-κB, and cleaved caspase-3,−8, and−9, promoting neuroprotection by attenuating oxidative stress induced apoptosis. Model type: *in vitro* (HT22 neurons, H_2_O_2_ injury). Sample size: cell replicates as reported. Evidence tier: preliminary (neuronal oxidative-stress model; no *in vivo* AD data) (Kim et al., 2020).

Durian (*Durio zibethinus*) seed polysaccharides (DSP), extracted via enzymatic hydrolysis and plasma treatment, yield a water-soluble polysaccharide (named DSPP-1), which is composed of galactose, glucuronic acid, and rhamnose. In transgenic *Caenorhabditis elegans* CL4176, an AD model, DSPP-1 reduced Aβ-induced toxicity and aggregation, lowered MDA levels, and enhanced antioxidant activity. These findings, supported by *in vitro* anti-Aβ aggregation and 2,2-diphenyl-1-picrylhydrazyl (DPPH) radical scavenging assays. Model types: *in vivo* (*C. elegans* CL4176) and *in vitro* (Aβ aggregation/DPPH assays). Sample size: NR; cell replicates as reported. Evidence tier: *preliminary* (non-mammalian *in vivo* + *in vitro* chemistry/biology) (Xiao et al., 2023).

## Polysaccharides modulating amyloid pathology and neuroprotective pathways

Several plant-derived polysaccharides directly influence amyloid pathology and associated pathways responsible for neuroprotection. *Astragalus membranaceus*, a flowering plant in TCM, contains Astragalus polysaccharide (APS) as a primary bioactive component, recognized for its neuroprotective and anti-inflammatory effects (Huang et al., 2017; Jia et al., 2019; Li et al., 2020). In Aβ42-transgenic *Drosophila melanogaster* models, APS delayed AD onset, extended lifespan, and improved motility by modulating Toll, IMD, and JAK-STAT signaling pathways. Specifically, the Toll and IMD pathways homologous to mammalian innate immune pathways, regulate inflammatory responses in the nervous system, while the JAK-STAT pathway mediates cytokine signaling and immune homeostasis. By modulating these pathways, APS reduces neuroinflammatory responses associated with amyloid toxicity resulting in enhanced neuronal survival, though it showed no benefit in tauopathy models. Model type: *in vivo* (*Drosophila* Aβ42 model). Sample size: NR. Evidence tier: preliminary (non-mammalian model with pathway modulation; no mammalian AD validation yet) (Li et al., 2023b).

*Codonopsis pilosula*, a perennial herb in the Campanulaceae, contains polysaccharides among its bioactive constituents, including saponins, alkaloids, and polyphenol glycosides, known for their protective effects against free radical damage and enhanced neuroprotection (Jiang et al., 2015; Weon et al., 2016; Fu et al., 2018; Hogan et al., 2019). In PC12 cells, *C. pilosula* polysaccharides mitigated Aβ-induced neurotoxicity by upregulating SIRT1, SIRT3, CD38, and PGC-1α expression (Hu et al., 2021c). SIRT1 and SIRT3 are NAD^+^-dependent deacetylases that regulate oxidative stress responses and mitochondrial function; however, CD38 modulates intracellular NAD^+^ levels influencing sirtuin activity, and PGC-1α acts as a transcriptional coactivator that enhances mitochondrial biogenesis and prevents formation of redox active compounds. Their upregulation collectively improves neuronal energy metabolism and reduces oxidative damage, contributing to neuroprotection. Model type: *in vitro* (PC12 neurons, Aβ injury). Sample size: cell replicates as reported. Evidence tier: preliminary (cell-based mechanisms) (Yan et al., 2022).

*Acanthopanax senticosus*, is another TCM herb, that contains polysaccharides alongside saponins, flavonoids, and coumarins, exhibiting potential to prevent damage to neurons. These polysaccharides mitigate Aβ-induced toxicity, reduce neuroinflammation, and enhance antioxidant activity, improving cognitive function by modulating neurotransmitter release, maintaining mitochondrial integrity, and regulating epigenetic pathways (Wang et al., 2022). In preclinical studies, *A. senticosus* polysaccharides attenuated apoptosis and prevented formation of redox active compounds, complementing the Nrf2-mediated effects of APS and the SIRT1/PGC-1α modulation of *Codonopsis pilosula* polysaccharides (Qin et al., 2020; Hu et al., 2021c).

## Bacterial polysaccharides and AD

Microbial exopolysaccharides (EPS) have shown potential for their neuroprotective potential in AD showing free radical scavenging, reduced inflammation, cholinesterase inhibition, and amyloid-modulating mechanisms in both *in vitro* and *in vivo* models. Evidence for bacterial polysaccharides is preliminary to moderate. Most findings derive from *in vitro* antioxidant and neuroinflammation assays, with a few supportive animal studies. No clinical data exists, and replication across laboratories is also limited.

## EPS targeting oxidative stress and neurotoxicity

Exopolysaccharides derived from *L. delbrueckii* sp. *bulgaricus* B3 and *Lactiplantibacillus plantarum* GD2 have been reported to protect SH-SY5Y neuronal cells from Aβ_1 − 42_-induced apoptosis by preserving mitochondrial membrane potential and reduction of oxidative damage. They have been reported to show free radical scavenging activity through DPPH radical scavenging, metal ion chelation, lipid peroxidation inhibition, and preservation of cellular redox balance. Model type: *in vitro* (SH-SY5Y cells, Aβ injury). Sample size: cell replicates. Evidence tier: preliminary (single-cell model studies without *in vivo* validation) (Sirin and Aslim, 2020, 2021). Additionally, lactic acid bacteria-derived exopolysaccharides (L-EPSs) modulate the nuclear factor erythroid 2-related factor 2/kelch-like ECH-associated protein 1 (NRF2-KEAP1) pathway in PC12 cells under H_2_O_2_-induced free radical formation and increase the expression of free radical scavenging enzymes (glutamate-cysteine ligase catalytic subunit (GCLC), HO-1, NQO1) as well as reduce ROS production. Model type: *in vitro* (PC12 neuronal cells, H_2_O_2_-induced stress). Sample size: cell replicates. Evidence tier: preliminary (mechanistic oxidative stress model; no animal studies yet) (Sirin, 2023).

## EPS modulating neuroinflammation and cholinesterase inhibition

EPS from *Marinobacter nauticus* strain GH3 (GH3-EPS) have multi-targeted neuroprotection through upregulation of antioxidant enzymes Glutathione peroxidase 4 (GPx-4), Glutathione synthetase (GSS), suppression of MDA and oxidative markers, inhibition of cyclooxygenase-2 (COX-2), and lipoxygenase (LOX) inflammatory pathways. They can achieve 52–68% acetylcholinesterase inhibition. *In vivo* fish models have shown GH3-EPS improved cognitive-like outcomes without toxicity which further supports its therapeutic relevance. Model types: *in vitro* (enzyme inhibition, free radical formation assays) and *in vivo* (fish behavioral model). Sample size: NR. Evidence tier: moderate (multi-target activity across cell and fish models; no mammalian AD validation yet) (Abdel-Razik et al., 2024).

## EPS reducing Aβ plaques and cognitive deficits in AD models

Recently, a structurally characterized exopolysaccharide from *Bacillus amyloliquefaciens* RK3 (composed of branched mannopyranose and galactopyranose units) has been reported to significantly reduce Aβ plaque deposition (through histopathological evidence) and improved cognitive function (through jumping box test) in male albino mice when administered at 200 mg/kg, and 400 mg/kg intraperitoneally. Moreover, acetylcholinesterase (AChE) inhibitory activity was also reported, suggesting a potential mechanism for enhancing cholinergic function. Specifically, this EPS was structurally characterized as a heteropolysaccharide composed of α-D-mannopyranose (α-D-Manp) and β-D-galactopyranose (β-D-Galp) units connected through specific glycosidic linkages (β-D-Galp(1 → 6)β-D-Galp(1 → 6)β-D-Galp(1 → 2)β-D-Galp(1 → 2)[β-D-Galp(1 → 6)]β-D-Galp(1 → 2)α-D-Manp(1 → 6)α-D-Manp(1 → 6)α-D-Manp(1 → 6)α-D-Manp(1 → 6)α-D-Manp) arranged in a branched tubular structure. Model type: *in vivo* (β-amyloid-induced AD in male albino mice), intraperitoneal 200–400 mg/kg. Sample size: 15 per group (5 groups). Evidence tier: moderate (single *in vivo* study with histological, biochemical, and behavioral endpoints) (Gangalla et al., 2021).

## Potential cholinesterase-inhibitory EPS from marine *Bacillus maritimus*

The EPS “BMEPS” derived from marine bacterium *Bacillus maritimus* strain (S16). It was reported that polysaccharide inhibited *ACh*E (IC_50_; 691.77 ± 8.65 μg/ml), and *BCh*E (IC_50_; 288.27 ± 10.50 μg/ml). Moreover, tyrosinase at IC_50_ activity was also inhibited at incubation durations of 10, 20, and 40 min. The other promising results include a selective anti-inflammatory action against COX-2, antioxidant capabilities, and ROS scavenging, metal chelation, and suppression of lipid peroxidation. These multidimensional effects show therapeutic potential against AD in *in vitro* studies; however, *in vivo* studies are still needed for confirmation of results and building on these potential results for future therapies. Model type: *in vitro* (enzyme inhibition, ROS assays). Sample size: cell replicates. Evidence tier: preliminary (mechanistic evidence only; no animal data yet) (Selim et al., 2023).

**Figure 6:**
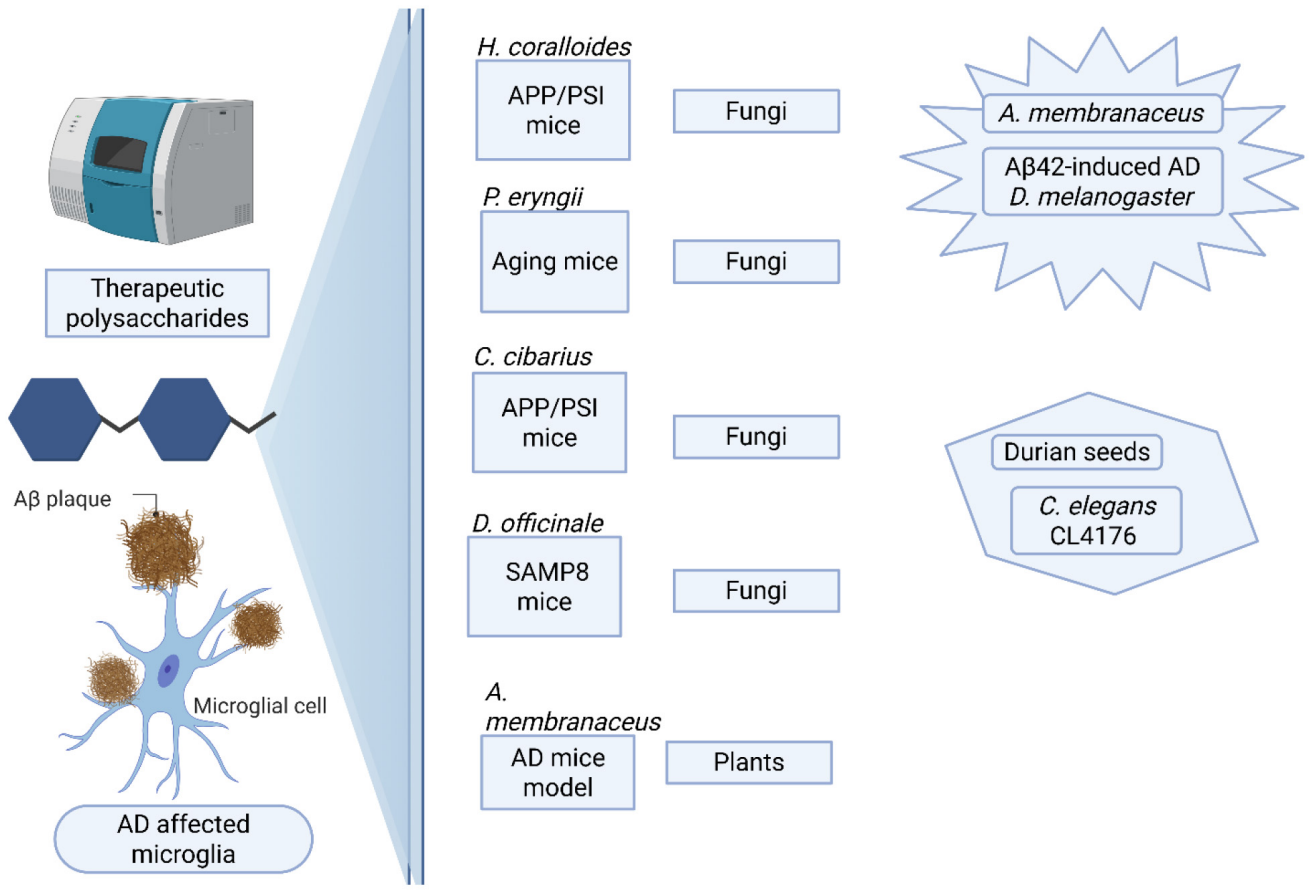
Schematic representation of *in vivo* studies showing sources and models used for exploring therapeutic potential of polysaccharides against AD.

Figure 6 illustrates *in vivo* studies of plant and fungal polysaccharides in AD models (e.g., SAMP8 mice for DOP, *C. elegans* for DSPP-1), highlighting fewer *in vivo* studies than *in vitro*, with limited algal *in vivo* data (e.g., SAMP8 mice for AOS). Further *in vivo* validation in AD-specific models (e.g., APP/PS1 mice) is essential to advance these polysaccharides' therapeutic potential.

## Conclusion

Natural polysaccharides from fungal, algal, plant, and bacterial sources hold significant promise as therapeutic agents for AD. These address multiple pathological features such as Aβ aggregation, tau hyperphosphorylation, neuroinflammation, oxidative stress, impaired autophagy, cholinergic dysfunction, and neuronal apoptosis. These polysaccharides modulate critical pathways such as Nrf2 activation, SIRT1 signaling, and autophagy, while inhibiting Aβ toxicity and microglial-driven inflammation in preclinical models like APP/PS1 mice and *Caenorhabditis elegans*. Unlike conventional therapies, which fail to stop progression of disease, polysaccharides offer multi-targeted, and low-toxicity interventions. However, their precise molecular mechanisms, bioavailability, and clinical efficacy remain underexplored. Future research should prioritize standardized extraction, and characterization methods, systematic pharmacokinetic and pharmacodynamic profiling, and multi-center clinical trials to validate efficacy and safety to accelerate clinical translation. Furthermore, the elucidation of structure-activity relationships will facilitate chemical modifications, and nanocarrier based delivery systems to enhance brain penetration, and therapeutic potential. To overcome translational barriers, coordinated efforts between academia, industry, and regulatory agencies are essential that will bring polysaccharide-based therapies closer to clinical applications against AD. The progress forwards will require close interdisciplinary collaboration across chemistry, pharmacology, and clinical research. Chemists can optimize structural modifications and standardization, pharmacologists can establish pharmacokinetics and safety profiles, and clinical researchers can design rigorous trials. Such integration is essential to translate polysaccharide-based therapeutics from promising preclinical data to clinical application in Alzheimer's disease.
